# Efficient *in vitro* assay for evaluating drug efficacy and synergy against emerging SARS-CoV-2 strains

**DOI:** 10.1128/aac.01233-24

**Published:** 2024-12-17

**Authors:** Maximillian Woodall, Samuel Ellis, Shengyuan Zhang, Japhette Kembou-Ringert, Kerry-Anne Kite, Laura Buggiotti, Amy I. Jacobs, Akosua Adom Agyeman, Tereza Masonou, Machaela Palor, Timothy D. McHugh, Judith Breuer, Joseph F. Standing, Claire M. Smith

**Affiliations:** 1 UCL Great Ormond Street Institute of Child Health 11700 , London, United Kingdom; 2 UCL Centre for Clinical Microbiology, Royal Free Campus 308349 , London, United Kingdom; 3 Department of Pharmacy, Great Ormond Street Hospital for Children 4956 , London, United Kingdom; Bill & Melinda Gates Medical Research Institute, Cambridge, Massachusetts, USA

**Keywords:** SARS-CoV-2, Omicron, COVID-19, infection, *in vitro* assay, drug synergy

## Abstract

Novel and repurposed antiviral drugs are available for the treatment of coronavirus disease 2019 (COVID-19). However, antiviral combinations may be more potent and lead to faster viral clearance, but the methods for screening antiviral combinations against respiratory viruses are not well established and labor-intensive. Here, we describe a time-efficient (72–96 h) and simple *in vitro* drug-sensitivity assay for severe acute respiratory syndrome coronavirus 2 (SARS-CoV-2) using standard 96-well plates. We employ different synergy models (zero interaction potency, highest single agent, Loewe, Bliss) to determine the efficacy of antiviral therapies and synergistic combinations against ancestral and emerging clinical SARS-CoV-2 strains. We found that monotherapy of remdesivir, nirmatrelvir, and active metabolite of molnupiravir (EIDD-1931) demonstrated baseline EC50s within clinically achievable levels of 4.34 mg/L (CI: 3.74–4.94 mg/L), 1.25 mg/L (CI: 1.10–1.45 mg/L), and 0.25 mg/L (CI: 0.20–0.30 mg/L), respectively, against the ancestral SARS-CoV-2 strain. However, their efficacy varied against newer Omicron variants BA.1.1.15 and BA.2, particularly with the protease inhibitor nirmatrelvir. We also found that remdesivir and nirmatrelvir have a consistent, strong synergistic effect (Bliss synergy score >10) at clinically relevant drug concentrations (nirmatrelvir 0.25–1 mg/L with remdesivir 1–4 mg/L) across all SARS-CoV-2 strains tested. This method offers a practical tool that streamlines the identification of effective combination therapies and the detection of antiviral resistance. Our findings support the use of antiviral drug combinations targeting multiple viral components to enhance COVID-19 treatment efficacy, particularly in the context of emerging viral strains.

## INTRODUCTION

Broad-spectrum antivirals approved for treating severe acute respiratory syndrome coronavirus 2 (SARS-CoV-2), such as remdesivir, molnupiravir, favipiravir, and nirmatrelvir, target key viral enzymes including RNA-dependent RNA polymerase (RdRp) and the main protease (Mpro or 3Cl protease) (1–5). While these drugs have proven largely clinically ineffective as monotherapies against coronavirus disease 2019 (COVID-19), combining these broad-spectrum antivirals, which target different stages of the virus’s replication cycle or host response, has shown promising *in vitro* efficacy and potential clinical benefits (6, 7). This approach follows the success of other combination therapies in treating viral infections like HIV and hepatitis C (8, 9). For instance, in immunocompromised patients, combinations of direct-acting antivirals (nirmatrelvir/ritonavir with molnupiravir (EIDD-2801), remdesivir, or a monoclonal antibody) were more effective than monotherapy, achieving sustained viral clearance in 85.4% of cases (10). Despite potential mutations in the viral genome, these broad-spectrum antivirals remain effective against various SARS-CoV-2 variants and other RNA viruses (including MERS-CoV, Ebola, influenza) (11–13), but continuous screening against newly emerging variants is essential to ensure the efficacy of existing treatments.

Phenotypic high-throughput screening, particularly assays that combine reporter cells and wild-type viruses (14), is an efficient method for identifying potential drug candidates (15) or repurposing clinically approved drugs (16). However, these assays face limitations with cross-lab standardization, crucial for ensuring data reproducibility. A notable instance of this issue was the prioritization of hydroxychloroquine for COVID-19 treatment. Initial reports suggested an EC50 of 0.24 mg/L for hydroxychloroquine (17), which was significantly lower (between 6- and 24-fold) than those reported by other groups (18). Despite this discrepancy, it led to the premature belief that hydroxychloroquine could be effective at clinically achievable concentrations, which it is not (19–21).

Standardization is vital, particularly regarding host cell types, time course, use of drug efflux inhibitors, addressing plate edge effects, and uniform statistical analysis, to improve the robustness and reproducibility of antiviral assays. Furthermore, the necessity for biosafety level 3 (BSL-3) containment presents additional challenges, especially in facilities that are not fully equipped for high-throughput research.

In this study, we develop and validate a time-efficient and simple method to quantify and analyze drug synergy enabling scalable high-throughput applications with other small molecules. We use this to identify the EC50 values for monotherapies and combinations of remdesivir, the active moiety of molnupiravir (EIDD-1931), nirmatrelvir, and favipiravir using clinically achievable drug ranges. The assay provides reproducible data across various clinical strains and is based on time-effective and simple technology and open-source software, making it feasible for both advanced and resource-limited facilities worldwide.

## MATERIALS AND METHODS

### Virus strains and cell lines

The SARS-CoV-2 isolate hCoV-19/England/2/2020 (classified as part of the Wuhan-Hu-1 lineage obtained from Public Health England, London) was used as the “ancestral” strain in this study. Clinical isolates of more recent SARS-CoV-2 variants were propagated from nasal swabs collected in a parallel virology study (22, 23). Specifically, we used three clinical SARS-CoV-2 isolates: AQ23 (BA.2 with L5F mutation), BD46 (BA.2), and AQ28 (BA.1.1.15). (22, 23). Whole-genome sequencing was performed as outlined in refs. (22, 23). Briefly, amplicon sequencing was performed with a target depth of 5,000× per genome on an Illumina sequencer using 2 × 150 bp paired-end reads. The entire processing of raw reads to consensus was carried out using nf-core/viralrecon pipeline (24).

African green monkey kidney cell line Vero E6 (ATCC: C1008-CRL-1586) was provided and authenticated by The Francis Crick Institute, London, UK, for use in this study. Vero E6 cells were maintained in Dulbecco’s Modified Eagle Medium (DMEM) supplemented with 5% fetal calf serum (Thermo Fisher) and 1× penicillin/streptomycin (Sigma-Aldrich). Media was replaced three times a week, and cells were maintained at 37°C and 5% CO_2_.

Calu-3 were purchased from ATCC (HTB-55 batch no.: 70042799) and maintained in DMEM supplemented with 5% fetal bovine serum (FBS) (Thermo Fisher) and 1× penicillin/streptomycin. Media was replaced three times a week, and cells were maintained at 37°C and 5% CO_2_.

### Viral propagation

For virus propagation, Vero E6 cells were infected with a multiplicity of infection (MOI) 0.01 PFU/cell, as performed previously (25, 26), in serum-free DMEM supplemented with 1% nonessential amino acids (Thermo Fisher), 0.3% (w/v) bovine serum albumin (Sigma), and 1× penicillin/streptomycin. The viruses were harvested after 48 h, aliquoted, and stored at −80°C.

### Cell seeding and preparation

**FIG 1:**
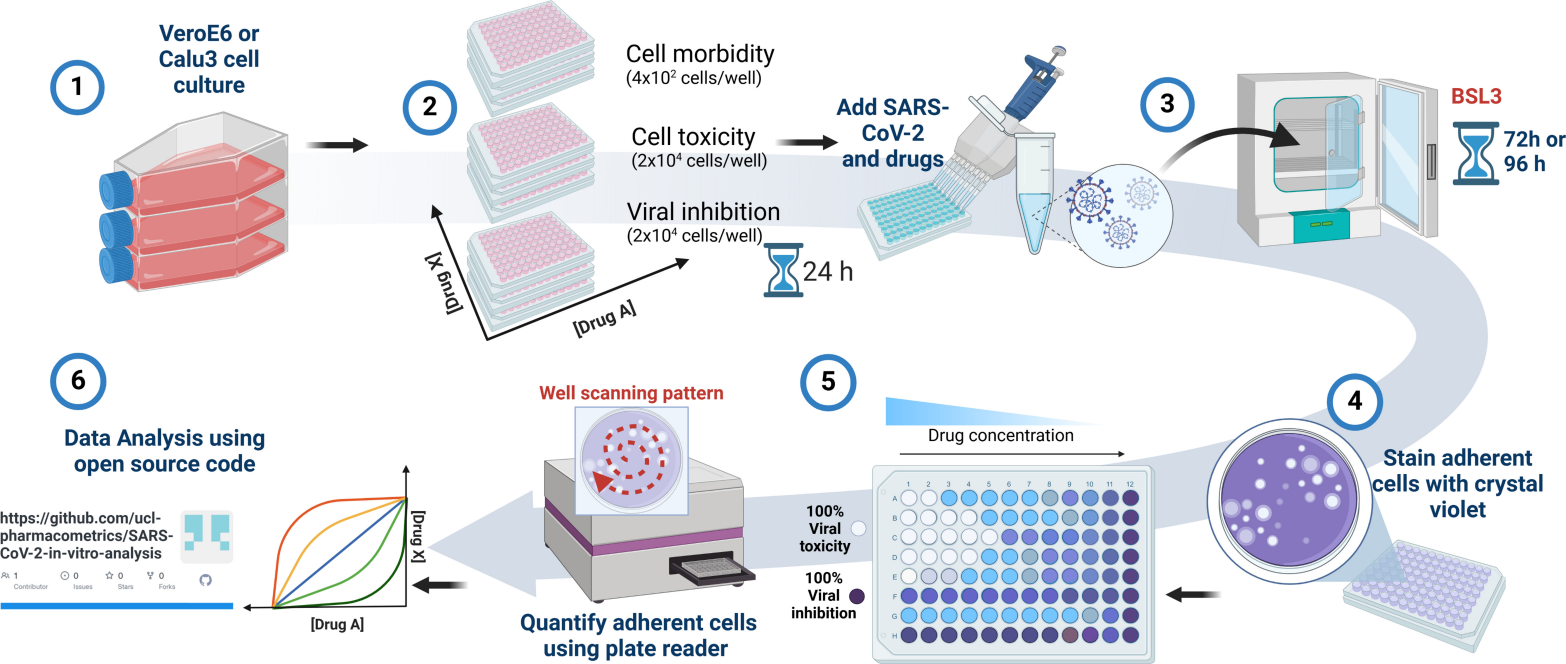
Schematic of method and experimental workflow used for standardizing the testing of monotherapy and combination therapies against SARS-CoV-2. (1) Vero E6 or Calu-3 cells are cultured at 37°C and 5% CO_2_. (2) Vero E6 or Calu-3 cells are seeded into 96-well plates (seeding density is given) and incubated overnight. (3) Cells are infected with SARS-CoV-2 (viral inhibition) or incubated with the drugs alone to test for cell toxicity or morbidity and incubated for 72 or 96 h. (4) After peak viral toxicity, adherent cells are fixed and stained with crystal violet. (5) Crystal violet staining is quantified by absorbance (595 nm) using a plate reader. (6) Data are analyzed using R. Graphic made using Biorender.com.

Cells were seeded on nine 96-well plates per experiment, with three replicate plates allocated for viral toxicity, cytotoxicity, and cytomorbidity assays, the three absorbance results from each plate were averaged per condition (Fig. 1). Plates allocated for cytotoxicity of the drugs and viral infection parameters were seeded with 2 × 10^4^ cells per well, in 100 µL of 5% v/v FBS media (Sigma). The remaining three plates were designated for cytomorbidity studies (inhibition of cell proliferation), with each plate receiving fewer cells per well to allow for expansion (4 × 10^2^) in 100 µL of 5% FBS media (27). Post-seeding, plates were agitated to ensure an even distribution of cells across the wells and incubated for 24 h at 37°C and 5% CO_2_.

### Drug plate preparation and infection protocol

**FIG 2:**
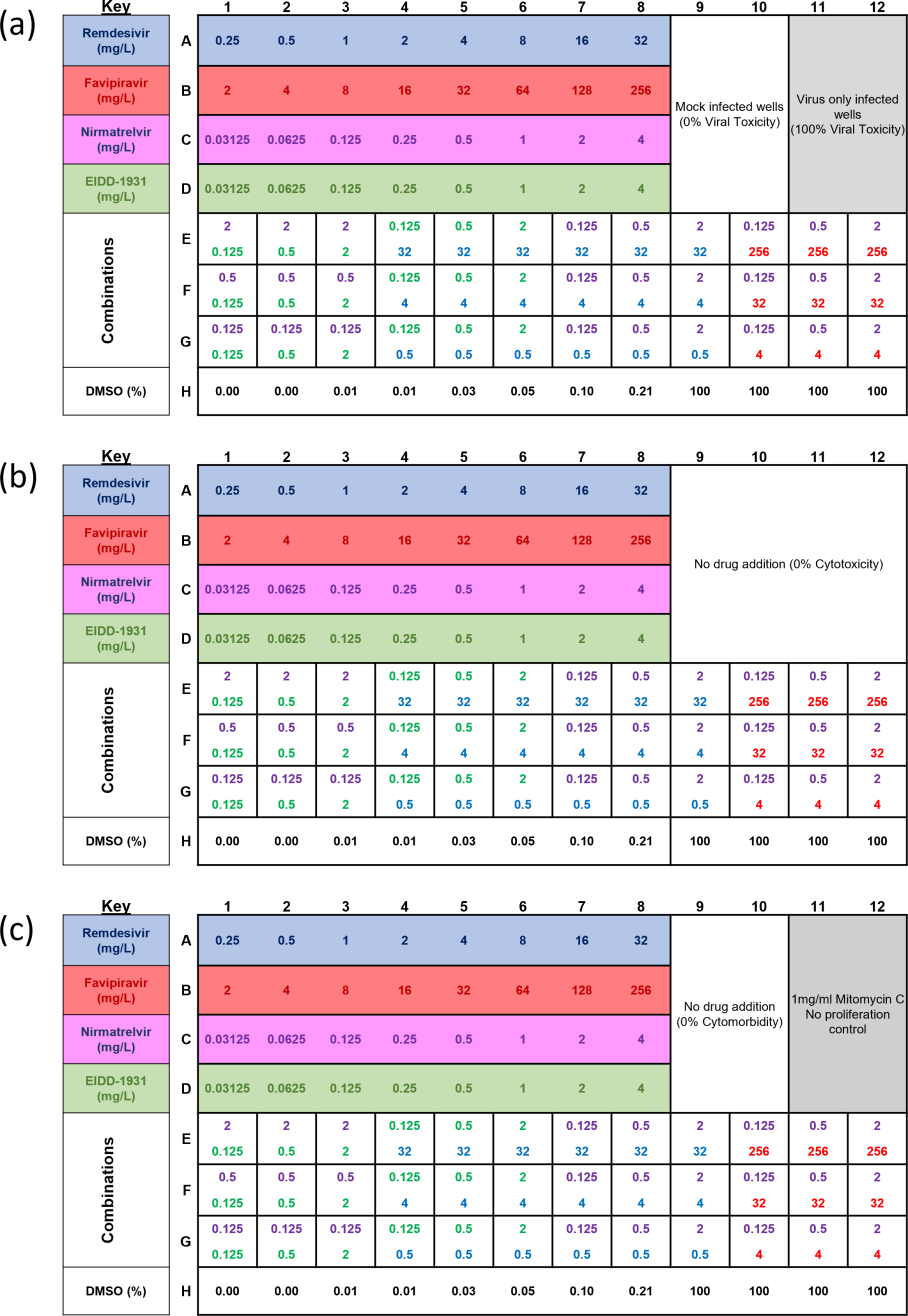
Example setup for a microplate assay. Example plate maps showing drug concentrations for monotherapies and combinations for each well of a 96-well plate. (**a**) Viral toxicity, (**b**) drug cytotoxicity, and (**c**) drug cytomorbidity.

Drug concentrations were chosen to fit within clinically achievable ranges (NIH COVID-19) (4, 22, 28, 28–31) and are detailed in the example drug distribution map (Fig. 2). A premade drug plate was prepared as an exact copy of the distribution shown in Fig. 2 but at 4× the final desired concentration of each drug. Then, 50 µL of the 4× drug solution was added from the premade drug plate to the appropriate wells of the 96-well plate containing the cells. The compounds used are as follows: remdesivir (Bio-Techne, Cat# 7226), favipiravir (Tocris Bioscience, Cat# 7225), EIDD-1931 (Sigma-Aldrich, SML2872), and nirmatrelvir (PF-07321332, Cambridge Bioscience, HY-138687). Each compound was solubilized in dimethyl sulfoxide (DMSO) (Sigma-Aldrich, 472301) at a stock concentration of 10 g/L and subsequently diluted in serum-free DMEM to the required concentrations. Stock solutions were stored at −20°C. Where indicated, we also added P-glycoprotein (Pgp) inhibitor CP-100356 at 1 mg/L (4× concentration) to the drug plate so that, when the cells were added, the final concentration in the well was 0.25 mg/L (50 µM). Next, 50 µL of 4× viral inoculum was added to the corresponding wells, resulting in a final MOI of 0.01. This MOI reflects the ratio of infectious viral particles to target cells, calculated based on an estimate of 2 × 10^4^ cells and 2 × 10^2^ plaque-forming units per well. An MOI of 0.01 was selected to enable multiple viral replication rounds, enhancing antiviral efficacy assessment and aligning with standard methodologies (27, 32–34). Control wells received either 50 µL of DMEM, 50 µL of 4× mitomycin C (4 mg/mL for final concentration of 1 mg/mL; Sigma-Aldrich, M4287), or 100% DMSO, to serve as uninfected controls, no proliferation (cytomorbidity) controls, and 100% cytotoxicity controls, respectively. Plates were incubated for 72 or 96 h at 37°C and 5% CO_2_.

### Post-infection processing and staining

Each well received 50 µL of a 5× fixative solution (15% w/v paraformaldehyde + 0.1% v/v crystal violet, PolySciences) and was left to incubate at room temperature for 30 min. Following incubation, the fixative solution was aspirated and discarded.

Wells were then washed three times with water and allowed to air dry. Once dried, absorbance at 595 nm was measured for each well using Spiral Averaging using FLUOstar Omega plate reader (BMG Labtech). Here, the plate reader takes 100 measurements for each well on a 6 mm spiral orbit and calculates an average. A schematic of the method is presented in Fig. 1.

### Antiviral statistical analysis

The dependent variable for the statistical analysis of antiviral effect was the percentage inhibition read from the optical density (35). Percent inhibition *I* for each well of a plate with *i* rows and *j* columns was calculated by


$$I_{ij}=(D_{ij}-D_{vc})/(D_{cc}-D_{vc})\times 100,$$

where *I*_*ij*_ is the percent inhibition in row *i* column *j*, *D*_*ij*_ the observed optical density from the well in row *i* column *j*, *D*_*vc*_ the mean optical density in the virus control wells, and *D*_*cc*_ the mean optical density in the cell control wells.

To account for the edge effect, plate mean normalization was applied, where each well’s signal was compared ith the mean signal of the entire plate before further analysis:

*D*_corrected_ = *D*_original_/*D*_blank_ where *D*_blank_ is the observed optical density from the same well from a blank plate.

This normalization step adjusts for systematic variations across the plate and was adapted from published methodologies (36, 37).

The Hill equation was then applied to this data estimating the predicted inhibition *P* as follows:


$$P_{ij}=E_{0}+E_{max}\;C_{ij}^{\lambda }\;/\;(EC50^{\lambda }+C_{ij}^{\lambda }),$$

where *P*_*ij*_ is the predicted viral inhibition in row *i* column *j*, *C*_*ij*_ is the drug concentration in row *i* column *j*, and *E*_0_, *E*_max_, EC50, and λ are the model parameters relating to the effect with no drug present, the maximum possible effect, the concentration required to elicit half the maximum effect, and the shape (Hill) parameter, respectively.

Eight possible models were fitted to each monotherapy data set, the simplest being with only EC50 estimated, *E*_0_ fixed to 0, *E*_max_ fixed to 100 and λ fixed to 1, and the most complex with all parameter estimated. Every possible combination of fixed and estimated parameters (with EC50 always estimated) was tested. Models were ranked based on Akaike information criteria (AIC), the model with the lowest AIC chosen. The nls function in R (version 4.3.2) was used.

### Synergy models for assessing drug combination efficacy

Synergy scores are calculated by R package SynergyFinder (38). Synergy score (*S*) is the change in observed drug response (*y*_comb_) compared with the noninteractive response (*y*_non-interaction_) defined by the model: *S* = *y*_comb_-*y*_non-interaction_

Given the distinct mechanisms of action—EIDD-1931 induces viral mutations, remdesivir terminates RNA chain extension, and nirmatrelvir inhibits protease—we give the Bliss independence model based on the assumption that the two drugs work independently: $y_{Bliss}=\;y_{1}+y_{2}-y_{1}.\;y_{2}$, where $y_{1},\;y_{2}$ represents the monotherapy drug response.

Loewe additivity (Loewe), highest single agent (HSA), and zero interaction potency (ZIP) models are the three other major synergy models (39, 40); we also provide these in the supplementary data for our study.

## RESULTS

### Assay optimization and standardization

**FIG 3:**
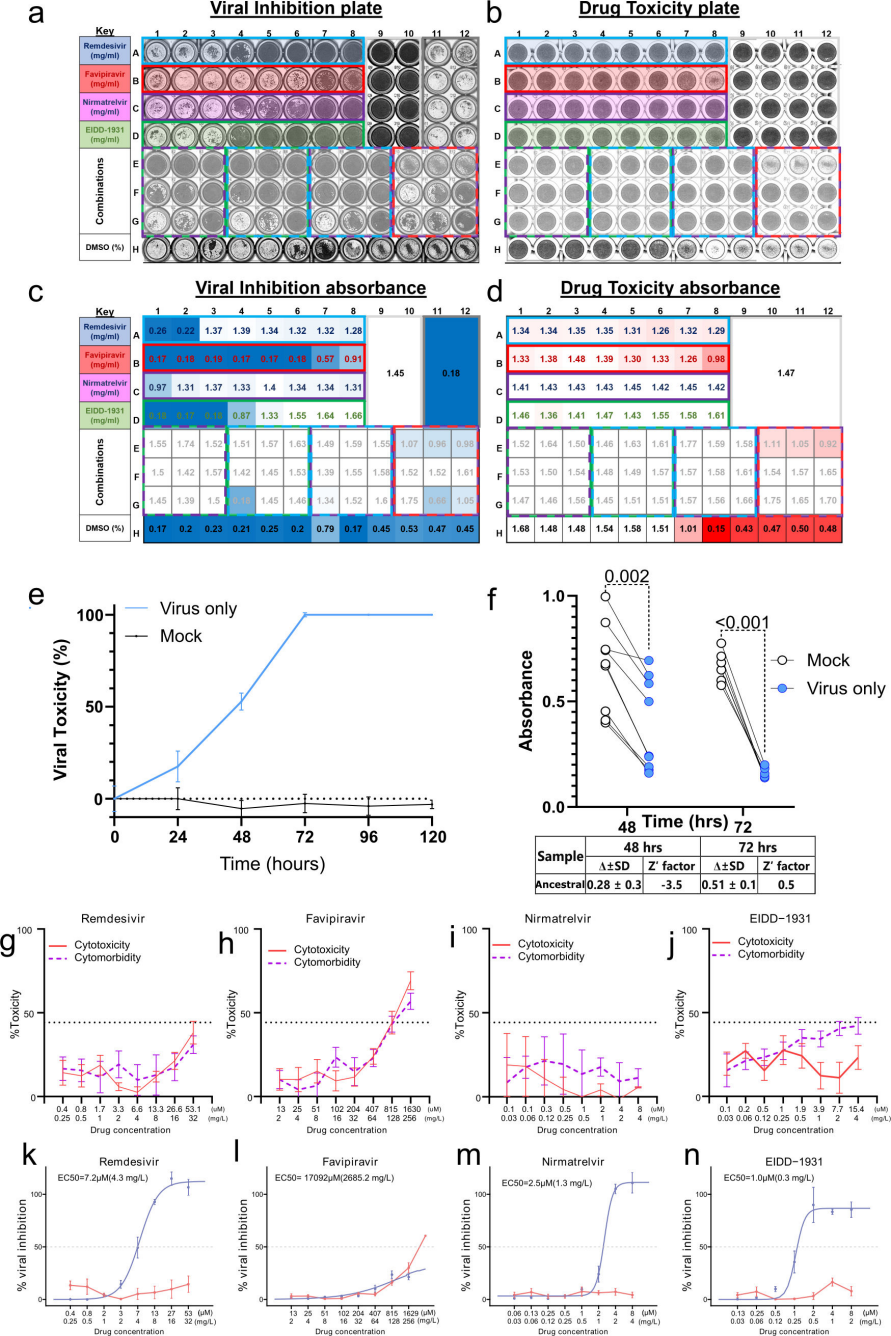
Antiviral efficacy and cytotoxicity of various compounds against ancestral SARS-CoV-2 strain in Vero E6 cell culture. (**a and b**) Representative image of a plate post-treatment and after staining with crystal violet for adherent viable cells for viral toxicity (**a**) and drug cytotoxicity (**b**). (**c and d**) Absorbance values at 595 nm quantified on a FLUOstar Omega plate reader, for viral toxicity (**c**) and drug cytotoxicity (**d**), with the color scale indicating red as the lowest absorbance (highest viral toxicity) and white as the highest absorbance (lowest viral toxicity). (**e**) Time course of viral toxicity for the ancestral strain against mock-infected wells, showing means ± SD. (**f**) Difference in absorbance at 595 nm for mock infected vs infected wells, at 48 h and 72 h post-infection time points, Z-factor for the assay at these time points is given in the table below. The dose-response curves for remdesivir (**g**), favipiravir (**h**), nirmatrelvir (**i**), and EIDD-1931 (**j**) showing % viral inhibition (blue lines) and cytotoxicity (red lines) at various drug concentrations. EC50 is given, and error bars represent the standard error (*n* = 6). The cytomorbidity-response curves at 72 h showing % cytotoxicity (solid red lines) and cytomorbidity (dashed red lines) at various drug concentrations for remdesivir (**k**), favipiravir (**l**), nirmatrelvir (**m**), and EIDD-1931 (**n**).

Our experimental design enables simultaneous testing of four individual drug dilutions and four combination treatments (Fig. 2) to assess potential synergy (Fig. 3a and b) as measured by the absorbance of crystal violet present in viable cells (Fig. 3c and d).

We found that peak viral toxicity for all strains tested occurred 72 h post-infection (mean ± SD: 48.3 ± 3.84 vs 86.5 ± 0.75 PFU/well; *n* = 6) (Fig. 3e). This time point provided the widest range for fitting the EC50 model and resulted in a desirable >0.5 Z-factor for reproducibility (absorbance difference 0.51 ± 0.7, *n* = 6) (Fig. 3f).

To control for potential false positives, caused by restricted cell growth in the absence of viral infection, we performed cytomorbidity assessments using the same drug concentrations (27). Here, we found that no drug concentration tested induced a cytomorbidity effect that reached >50% inhibition other than favipiravir (Fig. 3g through j), which also demonstrated a cytotoxicity >50% (CC50 219.47 mg/L) (Fig. 3d and h). We then set the limits of our model by first comparing fixed or estimated *E*_max_ and *E*_0_ (Fig. S1a through d) and adjusted our absorbance readings to account for the “edge effect” for more accurate and reproducible estimation of *E*_max_ and an optimal fit of the model (Fig. S1e through h).

Using an estimated *E*_max_ and λ (lowest AIC: Table S1) mode, we report EC50 (± SEM) values (Fig. 2k through n) of 4.34 ± 0.30 mg/L for remdesivir (Fig. 2k) and 1.25 ± 0.10 mg/L for nirmatrelvir (Fig. 3m). These values are >10-fold higher than those reported in other *in vitro* assays (1). However, we did not use a Pgp inhibitor to limit compound efflux in our assays, which could account for this difference. To investigate this further, we added a Pgp inhibitor (CP-100356 at 0.25 mg/L) and found that this reduced the EC50 of remdesivir 8-fold to 0.54 ± 0.042 mg/L (Fig. S2a). The EC50 of nirmatrelvir also reduced 25-fold in the presence of the Pgp inhibitor to 0.05 ± 0.045 mg/L (Fig. S2c). EIDD-1931 is not a substrate for Pgp (41), and therefore, the EC50 did not change in the presence of the Pgp inhibitor (0.25 ± 0.023 mg/L compared with 0.25 ± 0.025 mg/L) (Fig. S2d). Favipiravir proved ineffective as a viral inhibitor at all concentrations tested, similar to what others have found (16). The addition of the Pgp inhibitor did not alter the drug effectiveness of favipiravir (Fig. S2b).

Interestingly, the type of host cell used affected the potency of nirmatrelvir in the presence of Pgp inhibitor, enhancing its effectiveness by up to 10^6^-fold in Calu-3 cells (7.44 ± 0.10 mg/L to 0.025 ± 0.005 mg/L) compared to 1000-fold in Vero E6 cells (1.25 ± 0.10 mg/L to 0.050 ± 0.045 mg/L) (Fig. S3a through d; Table 1). While Pgp inhibitors are valuable tools in research, their use in clinical settings is limited, and there are concerns about the potential adverse effects due to the alteration of the pharmacokinetics of multiple drugs, leading to increased drug toxicity (42, 43). Therefore, we omitted their use in the rest of this study.

**TABLE 1:** EC50 values for each drug in different cell types with and without 0.5 µM CP-100356 efflux inhibitor

	Vero E6 EC50 ± SE (CI)		Vero + CP-100356 EC50 ± SE (CI)		Calu-3 EC50 ± SE (CI)		Calu-3 + CP-100356 EC50 ± SE (CI)	
	µM	mg/L	µM	mg/L	µM	mg/L	µM	mg/L
Remdesivir	7.20 ± 0.5 (6.2–8.2)	4.34 ± 0.3 (3.7–4.9)	0.85 ± 0.1 (0.7–1)	0.54 ± 0.0 (0.4–0.6)	1.53 ± 0.2 (1.2–1.9)	0.92 ± 0.1 (0.7–1.1)	1.24 ± 0.1 (1–1.5)	0.75 ± 0. (0.6–0.9)
Nirmatrelvir	2.50 ± 0.2 (2.2–2.9)	1.25 ± 0.1 (1.1–1.5)	0.09 ± 0.0 (0.1–0.1)	0.05 ± 0.0 (0.1–0.1)	14.84 ± 3.4 (8–22)	7.44 ± 0.1 (4.0–11)	0.05 ± 0 (0–0.1)	0.025 ± 0. (0–0.05)
EIDD-1931	0.89 ± 0.1 (0.6–1.1)	0.25 ± 0.0 (0.2–0.3)	1.00 ± 0.1 (0.7–1.3)	0.25 ± 0.0 (0.2–0.3)	1.19 ± 0.1 (1.1–1.3)	0.31 ± 0 (0.3–0.3)	1.23 ± 0.1 (1.1–1.4)	0.32 ± 0.0 (0.3–0.4)

We demonstrate that Calu-3 cells, a human airway cell line permissible to SARS-CoV-2 infection, can also be employed effectively in this assay allowing acquisition of reproducible EC50 values (Fig. S3a through d). However, we continued with Vero E6 cells as they are highly susceptible to all SARS-CoV-2 strains tested in our study, allowing for more direct comparison with previous research.

### Analysis of synergistic drug combinations against the ancestral strain of SARS-CoV-2

Our experimental setup allowed us to evaluate the effect of antiviral drugs combinations and determine synergy scores of up to four 2 × 2 drug combinations per run. We tested combinations of remdesivir and nirmatrelvir, remdesivir and EIDD-1931, and nirmatrelvir with EIDD-1931 (*n* = 6). Favipiravir was excluded from this analysis due to its high cytotoxic effects.

**FIG 4:**
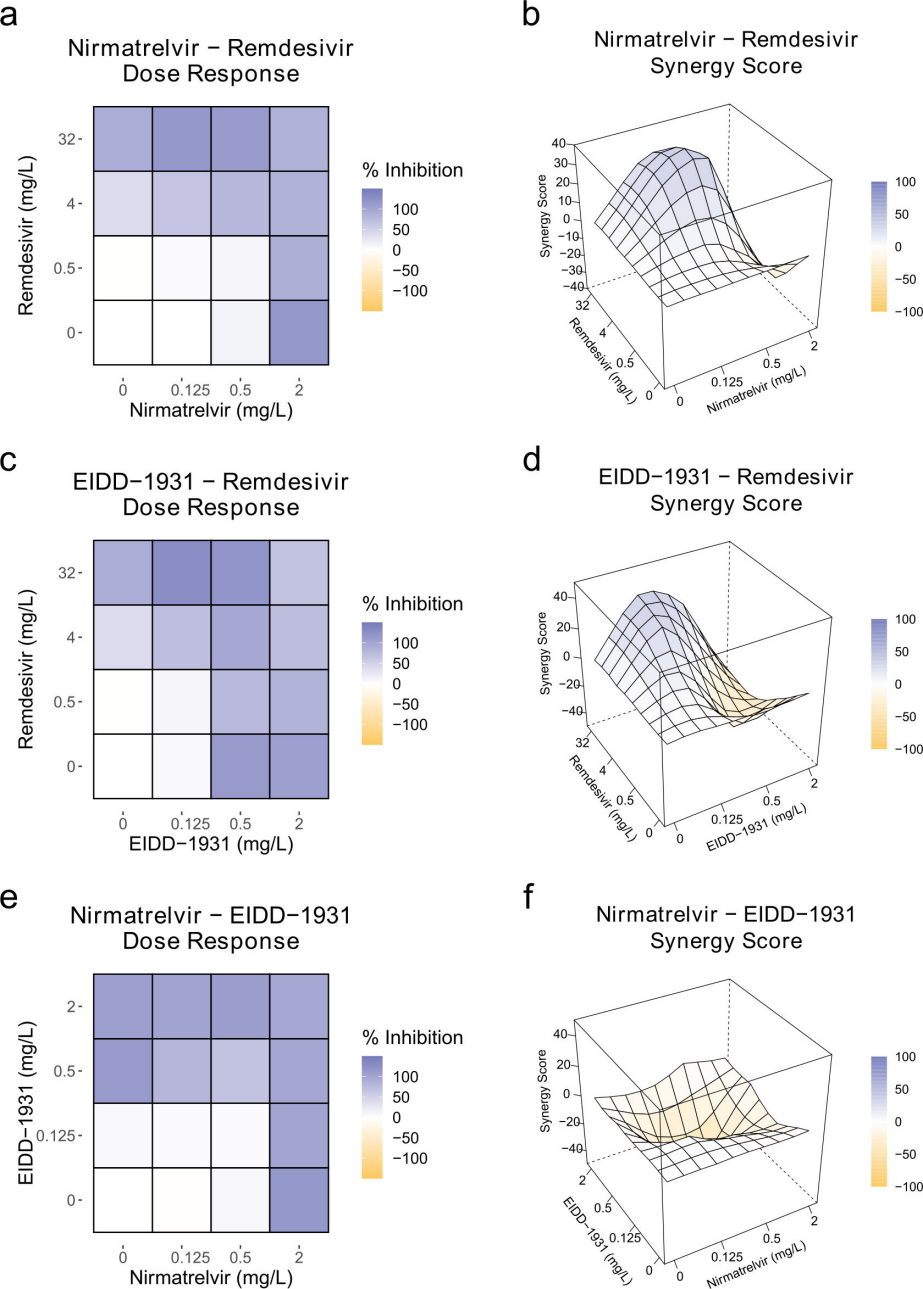
Antiviral drug combination efficacy against ancestral SARS-CoV-2 strain in Vero E6 cells. Heat maps represent viral inhibition and 3D matrices represent Bliss synergy scores for the same respective drug combinations for nirmatrelvir +remdesivir (**a and b**), nirmatrelvir +EIDD-1931 (**c and d**), and EIDD-1931 + remdesivir (**e and f**) combinations. Inhibition color scales represented as blue for high and white for low inhibition. Potential synergistic effects (0–100) are depicted in blue, no effect (=0) in white, and possible antagonistic effects (-100 to 0) in yellow. The color scales are given by the distance from 0. Showing mean scores (*n* = 6).

We found that the most synergistic drug combinations were 0.5 mg/L nirmatrelvir and 4 mg/L remdesivir, which achieved a Bliss synergy score of 32.6 (± 8.1, *n* = 6) (Fig. 4a and b) in Vero E6 cells and 43.7 (± 14.5, *n* = 3) in Calu-3 cells (Fig. S4; Table S2). This was supported by similar scores in the ZIP, HSA, and Loewe models, indicating consistent model agreement (Tables S2 and S3). Combining remdesivir (4 mg/L) with EIDD-1931 (0.125 mg/L) also yielded a synergistic Bliss score (29.25 ± 12.7, *n* = 6) (Fig. 4c and d), which was supported by most other synergy models (Table S2). In contrast, the combination of nirmatrelvir and EIDD-1931 only showed additive effects (Fig. 4e and f), with a Bliss score near zero (highest score of 5.1 ± 11 at 0.5 mg/L and 2 mg/L, *n* = 6), suggesting minimal interaction. Synergism of cytotoxic effects was minimal (<0 Bliss cytotoxicity synergy score), with only EIDD-1931 2 mg/L and remdesvir 4 mg/L showing a possible cytotoxicity increase (15.25 ± 8.31, *n* = 6), though this did not significantly influence synergy calculations (Table S5).

In conclusion, these synergy scores suggest that the combination of remdesivir and nirmatrelvir demonstrates enhanced antiviral efficacy against the ancestral strain of SARS-CoV-2 in Vero E6 cells, showing promise for potential therapeutic use. However, further investigation is needed to confirm this efficacy across other viral strains and cell lines.

### Testing monotherapy and drug combinations against emerging clinical isolates

**FIG 5:**
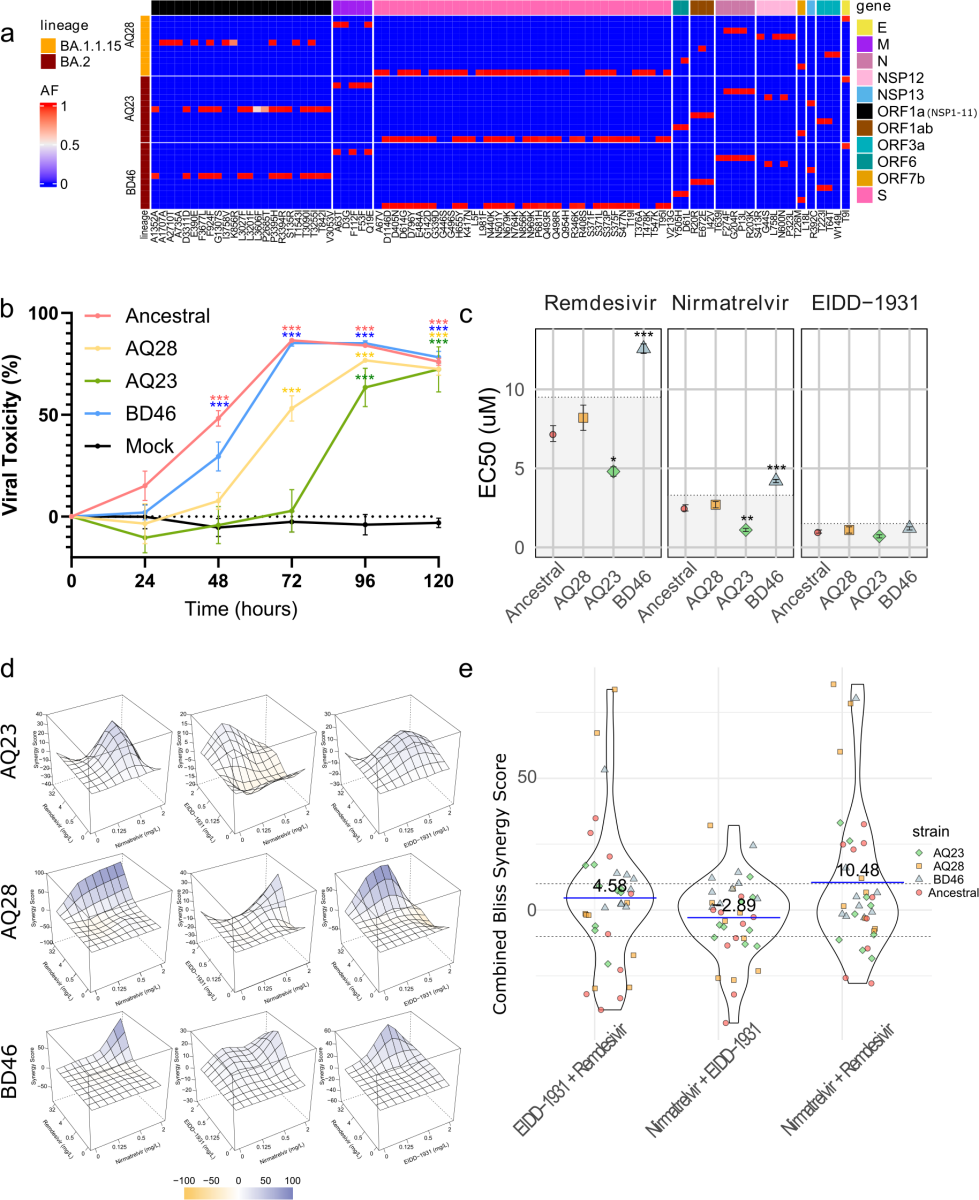
Characterization of antiviral drug efficacy and synergy across SARS-CoV-2 variants in Vero E6 cells. (**a**) Heat map showing the whole-genome landscape of consensus mutations within different clinical isolates (AQ28, AQ23, BD46) compared with the ancestral strain; allele frequency is represented by blue-red color scale. (**b**) Time course of viral toxicity generated by the ancestral, AQ28, AQ23, and BD46 viruses, showing means ± SD. Compared with mock-infected via ANOVA with Welch correction, using Dunnett’s multiple comparison test, (**c**) EC50 values for remdesivir, nirmatrelvir, and EIDD-1931 against AQ28, AQ23, and BD46 viruses, compared with the ancestral strain via ANOVA with Welch correction, using Dunnett’s multiple comparison test, *P*-values given. (**d**) 3D synergy maps showing Bliss synergy scores for drug combinations against AQ28, AQ23, and BD46 variants, where blue indicates high synergy, white indicates no effect, and yellow indicates antagonistic effects. (**e**) Violin plot of Bliss synergy scores for nirmatrelvir-remdesivir, nirmatrelvir-EIDD-1931, and EIDD-1931-remdesivir combinations, irrespective of concentration, for the different strains, with mean scores annotated. The color scale for both the heat map and synergy maps is provided. Statistical significance is indicated (*P < 0.05, **P < 0.01, ***P < 0.001).

We then tested the efficacy of these antiviral drugs against newly emerged SARS-CoV-2 clinical isolates with distinct viral genome sequences. All three isolates tested were Omicron variants (lineage BA 1.1.15 and BA 2), each presenting distinct, albeit similar, sequences (Fig. 5a). For instance, the AQ23 isolate (BA 2 + L5F) harbors distinct mutations from BD46 isolate (also BA 2) L3606F in NSP6, P2685T in NSP3, ntC26681T (F53F) in the M gene and L5F in the S gene (Fig. 5a).

Each isolate presented different time-dependent viral toxicity characteristics in Vero E6 cells, with the ancestral strain and BD46 (BA 2) generating significantly (*P* < 0.001 *n* = 6) more cell death at 72 h than AQ28 (BA 1.1.15) and AQ23 (BA 2) (Fig. 5b). However, all clinical isolates produced significant (*P* < 0.001) viral toxicity by 96 h when comparing infected and mock-infected wells (Fig. S5b). This time point also produced a higher assay reliability, as measured by Z’-factor (ancestral 0.8, AQ28 0.8, AQ23 0.3, BD46 0.8) (Fig. S5c) and was therefore selected for the drug-sensitivity assay.

We found that the EC50 of the drug monotherapies (remdesivir, nirmatrelvir, EIDD-1931, and favipiravir) remained fairly consistent across ancestral and clinical strains. Remdesivir showed a slight increase in sensitivity against AQ23 (EC50: 2.90 ± 0.35 mg/L, *P* < 0.05, *n* = 3) and a reduced activity against BD46 (EC50: 7.77 ± 0.47 mg/L, *P* < 0.001, *n* = 3) compared with the ancestral strain (EC50: 4.34 ± 1.21 mg/L, *n* = 3), with no changes against AQ28 (EC50: 4.94 ± 0.97 mg/L, *n* = 3) (Table S5d). Nirmatrelvir also displayed consistent efficacy, with AQ28 showing an EC50 of 1.35 ± 0.25 mg/L (ns, *n* = 3), a slight increase against AQ23 (EC50: 0.55 ± 0.10 mg/L, *P* < 0.001, *n* = 3) and a decrease against BD46 (EC50: 2.10 ± 0.08 mg/L, *n* = 3) compared with the ancestral strain (EC50: 1.25 ± 0.17 mg/L, *n* = 3) (Fig. S5e). EIDD-1931 maintained stable activity across all strains (EC50 ~0.25 mg/L, *n* = 3) (Fig. S5f). Favipiravir was ineffective against all strains (Fig. S5g). Given the experimental error range (CI ratios: remdesivir 1.322-fold, nirmatrelvir 1.318-fold, and molnupiravir 1.500-fold), only the slight increase in EC50 for BD46 with remdesivir and nirmatrelvir is noted, likely presenting little clinical relevance (Fig. 5c).

The combination of nirmatrelvir and remdesivir exhibited the highest synergistic effects across all isolates (Fig. 5d), with the optimal dose combination of 1 mg/L nirmatrelvir, 4 mg/L remdesivir, achieving a maximum synergy score of 85.7 ± 6.8 for AQ28 (*n* = 3–8), 80.4 ± 4.1 for BD46 (1 mg/L nirmatrelvir, 4 mg/L remdesivir, *n* = 3–8), 33.1 ± 6.3 for AQ23 (0.25 mg/L nirmatrelvir, 4 mg/L remdesivir, *n* = 3–8), and 32.6 ± 8.1 for the ancestral strain (0.5 mg/L nirmatrelvir, 4 mg/L remdesivir, *n* = 3–8). Drug combinations of nirmatrelvir + EIDD-1931 and EIDD-1931 + remdesivir showed limited synergistic activity across all strains, as indicated by scores close to or below zero without significant deviation from the ancestral strain. Combining all Bliss synergy scores for nirmatrelvir and remdesivir across all isolates and concentrations indicates a mean score of 10.48. This score (>10) represents a consistent synergistic mechanism that has been linked to improved therapeutic outcomes due to enhancing the potency of drug combinations (44) (Fig. 5e).

## DISCUSSION

Here, we describe a simple *in vitro* drug-sensitivity assay for SARS-CoV-2 that we used to determine the efficacy of antiviral therapies and synergistic combinations against both ancestral and newer SARS-CoV-2 strains obtained from clinical samples. Importantly, as indicated below, we describe the steps we have taken to standardize our assay and make code freely available for comparative analysis.

First, we included a cytomorbidity assay to test for potential false positives, aligning with recent practices (27). Second, we used different SARS-CoV-2 clinical isolates and selected time points post-infection, resulting in complete cell death as key indicators for measuring drug efficacy and synergy, improving assay reproducibility, and establishing reliable measurement standards. Finally, we show this assay is applicable for both Vero E6 and Calu-3 cells and show the impact of cellular model selection for antiviral testing, including the use of Pgp inhibitors.

Further refinements, such as addressing the plate edge effect (45) and estimating 𝐸_max_ in our model fit, led to minor modifications in the EC50s of monotherapy across SARS-CoV-2 isolates, but will contribute to data quality and consistency. We applied plate mean normalization to account for edge effects, a method adapted from published methodologies (36, 37). This normalization step ensures more reliable results by mitigating systematic variations across the plate, ultimately enhancing the robustness of antiviral efficacy assessments.

We observed differences in drug effectiveness between Vero E6 and Calu-3 cells. Both cell types express the Pgp efflux pump (MDR1/ABCB1), which often requires a Pgp inhibitor to prevent compound export and can affect antiviral activity (46, 47). These differences highlight the impact of cellular model selection and Pgp susceptibility. Other models are being developed, such as engineered A549 and H1299 human cell lines with exogenous receptor expression show high susceptibility to SARS-CoV-2 variants (48, 49) and VeroE6-Pgp-KO allows for control of Pgp activity (50). The incorporation of such physiologically relevant cell lines could enhance the accuracy of pre-clinical drug testing within this assay. Importantly, our assay design permitted the effective use of Calu-3 cells, demonstrating its versatility and potential applicability to other cell models.

Indeed, our results are in line with similar, but more labor-intensive work using a secondary plaque assay following initial drug exposure (51). This work reported similar monotherapy EC50 values for remdesivir, nirmatrelvir, and molnupiravir in Calu-3 cells with a Pgp efflux inhibitor as our study (e.g., 0.26 mg/L, 0.047 mg/L, and 0.012 mg/L, respectively) (51). Both studies also achieved similar peak Bliss synergy scores (~30) for nirmatrelvir and remdesivir combinations, demonstrating the robustness and efficiency of our simpler streamlined approach. Additionally, emerging methods like the two-way pharmacodynamic model that may more accurately assess such drug combination synergy at these clinically untested concentrations can aid in improving cross-study comparisons and data integration (52).

In terms of drug efficacy, our results indicate that SARS-CoV-2 strains may have different susceptibilities to remdesivir and nirmatrelvir as monotherapies, with certain mutations like those present in the BD46 isolate (BA.2) potentially decreasing sensitivity to antivirals. This response is consistent with recent work showing increased median remdesivir and nirmatrelvir EC50s (0.75 mg/L and 0.28 mg/L, respectively) compared with a similar reference strain (hCoV/Korea/KCDC03/2020; >99.5% sequence similarity to the ancestral strain) (53). While this may have little clinical relevance immediately, it highlights the complexity of reporting drug response across different SARS-CoV-2 variants/isolates and underscores the need for more research to monitor the development of drug resistance due to viral mutations (1, 22, 23). Despite potential mutations in the viral genome that may confer partial resistance (e.g., remdesivir: E796D, E802D; nirmatrelvir: S144A, E166V) (54–58), broad-spectrum antivirals can remain effective as combination therapies. To this point, it is essential to have rapid and accurate *in vitro* assays to detect the development of antiviral resistance as part of a global strategy for viral outbreak preparedness (59).

Combination therapies, targeting different parts of the viral replication cycle, can offer an effective solution to these issues (51, 60–64). Our data show that combined remdesivir and nirmatrelvir demonstrated a consistently strong synergistic effect across all strains. High synergy scores > 10, such as those derived from Bliss independence models, have been linked to improved therapeutic outcomes as they reflect enhanced potency of drug combinations (44). This is especially important in antiviral therapies, where studies have shown that synergistic drug interactions can lead to more efficient viral suppression and improved patient recovery times compared with monotherapy (6, 10, 61). While we observe some antagonistic interactions between these drugs at high concentrations, this does not undermine their clinical usefulness, as seen with effective HIV therapies (65). This aligns with work demonstrating that remdesivir and nirmatrelvir have significant synergistic activity (HSA > 10) against the 20A.EU1 strain, which translated to positive clinical outcomes in an immunocompromised severe COVID-19 patient (61). This suggests that targeting multiple viral components, such as Mpro and RdRp (66), with protease inhibitors like nirmatrelvir and viral polymerase inhibitors like remdesivir, may maintain efficacy over time, despite emerging variants, especially as resistance mutations have not become predominant in the viral population (67). Indeed an ever-expanding list of effective SARS-CoV-2 viral protease inhibitors (2, 63, 68) and RNA polymerase inhibitors (64, 69, 70) may fit this strategy.

In conclusion, our study presents a time-efficient method for evaluating the efficacy of broad-spectrum antiviral drugs, both as monotherapies and in combination. We demonstrate the benefits of using synergistic drug combinations against various SARS-CoV-2 variants. With the continual evolution of the virus, ongoing efficacy testing and early resistance monitoring are essential. This method offers a practical tool that aids the identification of effective combination therapies and detection of antiviral resistance, better equipping researchers to address the evolving challenges of COVID-19 treatment.

## Data Availability

Source data for the numerical figures in this study are provided at https://doi.org/10.6084/m9.figshare.27187479.v1. Custom code for the analysis performed in this study is publicly available at https://github.com/ucl-pharmacometrics/SARS-CoV-2-in-vitro-analysis.

## SUPPLEMENTAL MATERIAL

Supplemental material aac.01233-24-s0001.docx Tables S1 to S4 and Figures S1 to S5.

ASM does not own the copyrights to Supplemental Material that may be linked to, or accessed through, an article. The authors have granted ASM a non-exclusive, world-wide license to publish the Supplemental Material files. Please contact the corresponding author directly for reuse.
