# Supplementary material for: Efficient in vitro assay for evaluating drug efficacy and synergy against emerging SARS-CoV-2 strains
Source: Antimicrob Agents Chemother. 2024 Dec 17;69(2):e01233-24. doi: 10.1128/aac.01233-24 (PMC11823597; doi:10.1128/aac.01233-24)
Supplement: Supplemental material — Tables S1 to S4 and Figures S1 to S5. [file aac.01233-24-s0001.docx]

Supplemental Materials

**Supplemental Tables S1-S4**

**Supplemental Table S1.** The Akaike information criterion (AIC) for models applied to dose response curves.

| Drug | Model | Description | AIC |
| --- | --- | --- | --- |
| Remdesivir | m1 | estimating only EC50 | 440.18 |
| Remdesivir | m2 | estimating EC50 and λ | 391.34 |
| Remdesivir | m3 | estimating EC50 and E_max_ | 416.01 |
| Remdesivir | m4 | estimating EC50 and E0 | 442.12 |
| Remdesivir | m5 | estimating EC50, E_max_ and E0 | 404.32 |
| Remdesivir | m6 | estimating EC50, E_max_ and λ | 383.7 |
| Remdesivir | m7 | estimating EC50, E0 and λ | 390.24 |
| Remdesivir | m8 | estimating EC50, E_max_, E0, and λ | 385.41 |
| Favipiravir | m1 | estimating only EC50 | 352.75 |
| Favipiravir | m2 | estimating EC50 and λ | 340.54 |
| Favipiravir | m3 | estimating EC50 and E_max_ | 331.4 |
| Favipiravir | m4 | estimating EC50 and E0 | 351.54 |
| Favipiravir | m5 | estimating EC50, E_max_ and E0 | 331.75 |
| Favipiravir | m6 | estimating EC50, E_max_ and λ | fail |
| Favipiravir | m7 | estimating EC50, E0 and λ | 337.44 |
| Favipiravir | m8 | estimating EC50, E_max_, E0, and λ | fail |
| Nirmatrelvir | m1 | estimating only EC50 | 449.83 |
| Nirmatrelvir | m2 | estimating EC50 and λ | fail |
| Nirmatrelvir | m3 | estimating EC50 and E_max_ | 419.94 |
| Nirmatrelvir | m4 | estimating EC50 and E0 | 451.5 |
| Nirmatrelvir | m5 | estimating EC50, E_max_ and E0 | 415.87 |
| Nirmatrelvir | m6 | estimating EC50, E_max_ and λ | 377.7 |
| Nirmatrelvir | m7 | estimating EC50, E0 and λ | fail |
| Nirmatrelvir | m8 | estimating EC50, E_max_, E0, and λ | 379.07 |
| EIDD-1931 | m1 | estimating only EC50 | 445.85 |
| EIDD-1931 | m2 | estimating EC50 and λ | 438.74 |
| EIDD-1931 | m3 | estimating EC50 and E_max_ | 447.71 |
| EIDD-1931 | m4 | estimating EC50 and E0 | 443.95 |
| EIDD-1931 | m5 | estimating EC50, E_max_ and E0 | 442.23 |
| EIDD-1931 | m6 | estimating EC50, E_max_ and λ | fail |
| EIDD-1931 | m7 | estimating EC50, E0 and λ | 433.21 |
| EIDD-1931 | m8 | estimating EC50, E_max_, E0, and λ | fail |

**Supplemental Table S2**. Synergy scores for different drug combinations against ancestral SARS-CoV-2 strain in Calu-3 cells.

| **Combination** | **conc1** | | **conc2** | | **Bliss score** | | **Bliss sd** | | **Loewe score** | | **Loewe sd** | | **HSA score** | | **HSA sd** | | **ZIP score** | | **ZIP sd** |
| --- | --- | --- | --- | --- | --- | --- | --- | --- | --- | --- | --- | --- | --- | --- | --- | --- | --- | --- | --- |
| Nirmatrelvir - Remdesivir | 0.125 | | 32 | | 23.0 | | 7.1 | | 23.3 | | 7.0 | | 23.0 | | 7.2 | | 20.4 | | 5.8 |
| Nirmatrelvir - Remdesivir | 0.5 | | 32 | | 15.3 | | 4.8 | | 17.9 | | 6.3 | | 16.9 | | 5.5 | | 15.7 | | 4.5 |
| Nirmatrelvir - Remdesivir | 2 | | 32 | | 17.7 | | 5.5 | | 16.4 | | 17.4 | | 16.6 | | 18.2 | | 13.7 | | 8.0 |
| Nirmatrelvir - Remdesivir | 0.125 | | 4 | | 42.6 | | 6.0 | | 21.9 | | 16.0 | | 44.5 | | 6.0 | | 43.2 | | 6.4 |
| Nirmatrelvir - Remdesivir | 0.5 | | 4 | | 43.7 | | 14.5 | | 21.7 | | 15.6 | | 54.8 | | 12.5 | | 45.2 | | 10.4 |
| Nirmatrelvir - Remdesivir | 2 | | 4 | | 37.2 | | 24.5 | | 19.6 | | 24.4 | | 46.1 | | 32.7 | | 32.3 | | 23.0 |
| Nirmatrelvir - Remdesivir | 0.125 | | 0.5 | | -2.3 | | 17.2 | | -2.6 | | 8.5 | | -1.3 | | 10.3 | | 2.0 | | 3.9 |
| Nirmatrelvir - Remdesivir | 0.5 | | 0.5 | | -2.4 | | 31.4 | | -8.7 | | 30.7 | | -3.0 | | 29.4 | | 7.0 | | 16.2 |
| Nirmatrelvir - Remdesivir | 2 | | 0.5 | | 25.1 | | 52.2 | | 8.9 | | 44.7 | | 25.2 | | 52.5 | | 22.3 | | 43.6 |
| Nirmatrelvir - EIDD-1931 | 2 | | 0.125 | | 11.0 | | 46.5 | | 12.4 | | 46.8 | | 11.9 | | 47.3 | | 16.3 | | 44.0 |
| Nirmatrelvir - EIDD-1931 | 2 | | 0.5 | | 50.3 | | 37.0 | | 28.9 | | 40.4 | | 56.3 | | 42.2 | | 45.2 | | 37.4 |
| Nirmatrelvir - EIDD-1931 | 2 | | 2 | | 0.0 | | 3.8 | | -6.0 | | 13.6 | | -6.0 | | 13.8 | | 1.6 | | 4.1 |
| Nirmatrelvir - EIDD-1931 | 0.5 | | 0.125 | | 13.6 | | 19.7 | | 8.6 | | 21.0 | | 15.1 | | 16.7 | | 16.6 | | 14.3 |
| Nirmatrelvir - EIDD-1931 | 0.5 | | 0.5 | | 56.8 | | 13.2 | | 45.3 | | 24.6 | | 65.2 | | 8.3 | | 57.9 | | 11.9 |
| Nirmatrelvir - EIDD-1931 | 0.5 | | 2 | | 0.2 | | 3.4 | | -1.5 | | 5.6 | | -0.5 | | 4.4 | | -0.4 | | 3.2 |
| Nirmatrelvir - EIDD-1931 | 0.125 | | 0.125 | | -4.2 | | 16.5 | | -4.7 | | 7.5 | | -2.9 | | 9.6 | | 1.0 | | 0.7 |
| Nirmatrelvir - EIDD-1931 | 0.125 | | 0.5 | | 46.5 | | 11.6 | | 38.7 | | 14.0 | | 47.6 | | 9.3 | | 46.3 | | 11.1 |
| Nirmatrelvir - EIDD-1931 | 0.125 | | 2 | | -0.8 | | 5.0 | | -2.1 | | 5.3 | | -1.1 | | 5.1 | | -0.9 | | 4.1 |
| EIDD-1931 - Remdesivir | 0.125 | | 32 | | 7.5 | | 7.0 | | 6.1 | | 7.9 | | 7.5 | | 7.0 | | 8.0 | | 6.3 |
| EIDD-1931 - Remdesivir | 0.5 | 32 | | 19.4 | | 7.5 | | 12.6 | | 7.2 | | 20.5 | | 8.7 | | 15.0 | | 5.9 | |
| EIDD-1931 - Remdesivir | 2 | 32 | | 0.9 | | 2.9 | | -3.2 | | 5.5 | | -3.1 | | 5.2 | | 3.7 | | 2.7 | |
| EIDD-1931 - Remdesivir | 0.125 | 4 | | 31.6 | | 8.3 | | 10.7 | | 9.6 | | 32.0 | | 8.4 | | 31.4 | | 8.1 | |
| EIDD-1931 - Remdesivir | 0.5 | 4 | | 38.2 | | 3.9 | | 4.4 | | 4.9 | | 45.7 | | 3.1 | | 38.7 | | 4.0 | |
| EIDD-1931 - Remdesivir | 2 | 4 | | 4.5 | | 5.2 | | 1.8 | | 5.2 | | 2.7 | | 6.3 | | 3.6 | | 4.3 | |
| EIDD-1931 - Remdesivir | 0.125 | 0.5 | | 2.4 | | 0.6 | | -1.8 | | 1.9 | | 0.4 | | 0.6 | | 1.4 | | 0.4 | |
| EIDD-1931 - Remdesivir | 0.5 | 0.5 | | 48.1 | | 14.1 | | 31.5 | | 17.1 | | 46.3 | | 14.0 | | 47.1 | | 14.0 | |
| EIDD-1931 - Remdesivir | 2 | 0.5 | | 3.2 | | 5.6 | | 2.3 | | 3.6 | | 3.2 | | 5.6 | | 2.6 | | 4.6 | |
| Favipiravir - Nirmatrelvir | 256 | 0.125 | | 9.7 | | 8.8 | | 12.0 | | 9.3 | | 12.0 | | 7.5 | | 10.5 | | 6.3 | |
| Favipiravir - Nirmatrelvir | 256 | 0.5 | | 0.1 | | 17.6 | | 13.7 | | 13.8 | | 13.2 | | 12.8 | | -0.5 | | 14.8 | |
| Favipiravir - Nirmatrelvir | 256 | 2 | | -17.1 | | 23.9 | | -6.5 | | 28.7 | | -7.9 | | 27.7 | | -11.6 | | 21.0 | |
| Favipiravir - Nirmatrelvir | 32 | 0.125 | | -2.7 | | 11.8 | | -10.2 | | 6.5 | | -2.5 | | 11.4 | | -0.6 | | 7.7 | |
| Favipiravir - Nirmatrelvir | 32 | 0.5 | | 33.2 | | 25.7 | | 18.1 | | 23.2 | | 34.1 | | 24.9 | | 29.8 | | 22.3 | |
| Favipiravir - Nirmatrelvir | 32 | 2 | | 45.0 | | 37.6 | | 43.8 | | 35.7 | | 46.2 | | 38.6 | | 33.9 | | 27.2 | |
| Favipiravir - Nirmatrelvir | 4 | 0.125 | | 5.0 | | 21.7 | | 6.9 | | 9.1 | | 5.9 | | 13.8 | | 5.2 | | 3.2 | |
| Favipiravir - Nirmatrelvir | 4 | 0.5 | | 37.8 | | 25.8 | | 32.1 | | 22.3 | | 38.2 | | 22.4 | | 33.5 | | 21.7 | |
| Favipiravir - Nirmatrelvir | 4 | 2 | | 42.5 | | 30.8 | | 43.2 | | 30.1 | | 43.1 | | 30.5 | | 35.4 | | 24.9 | |

**Supplemental Table S3**. Synergy scores for different drug combinations across SARS-CoV-2 strains.

| **strain** | **drug1** | **drug2** | **conc1** | **conc2** | **Bliss score** | **Bliss sd** | **Loewe score** | **Loewe sd** | **HSA score** | **HSA sd** | **ZIP score** | **ZIP sd** |
| --- | --- | --- | --- | --- | --- | --- | --- | --- | --- | --- | --- | --- |
| Wuhan | EIDD-1931 | Remdesivir | 0.125 | 0.5 | 6.2 | 5.3 | -8.1 | 14.4 | 5.4 | 5.2 | 5.8 | 4.8 |
| Wuhan | EIDD-1931 | Remdesivir | 0.125 | 4 | 29.2 | 12.7 | -15.8 | 21.5 | 31.9 | 13.3 | 26.4 | 10.6 |
| Wuhan | EIDD-1931 | Remdesivir | 0.125 | 32 | 34.8 | 21.9 | 17.2 | 17.7 | 35.1 | 22.4 | 28.6 | 16.3 |
| Wuhan | EIDD-1931 | Remdesivir | 0.5 | 0.5 | -31.9 | 24.1 | -28.0 | 23.3 | -31.8 | 24.1 | -20.2 | 14.6 |
| Wuhan | EIDD-1931 | Remdesivir | 0.5 | 4 | -9.1 | 14.5 | -11.2 | 13.4 | -15.0 | 14.5 | -14.7 | 9.0 |
| Wuhan | EIDD-1931 | Remdesivir | 0.5 | 32 | 20.3 | 5.6 | 9.3 | 5.3 | 6.6 | 6.4 | 12.2 | 7.0 |
| Wuhan | EIDD-1931 | Remdesivir | 2 | 0.5 | -22.7 | 11.3 | -25.9 | 9.4 | -22.7 | 11.3 | -22.9 | 10.9 |
| Wuhan | EIDD-1931 | Remdesivir | 2 | 4 | -37.9 | 7.2 | -44.6 | 7.0 | -41.4 | 8.4 | -34.9 | 6.8 |
| Wuhan | EIDD-1931 | Remdesivir | 2 | 32 | -33.5 | 3.3 | -45.0 | 2.7 | -42.3 | 4.6 | -25.6 | 7.9 |
| BD46 | EIDD-1931 | Remdesivir | 0.03125 | 0.25 | 2.1 | 0.4 | -0.6 | 0.4 | 0.1 | 0.4 | 0.0 | 0.2 |
| BD46 | EIDD-1931 | Remdesivir | 0.03125 | 1 | 0.8 | 1.2 | -5.0 | 1.2 | -1.2 | 1.2 | 0.5 | 0.7 |
| BD46 | EIDD-1931 | Remdesivir | 0.03125 | 4 | 13.3 | 3.0 | 11.1 | 3.0 | 11.4 | 3.0 | 12.2 | 3.0 |
| BD46 | EIDD-1931 | Remdesivir | 0.125 | 0.25 | 1.2 | 0.6 | -2.9 | 0.6 | 1.3 | 0.6 | 0.4 | 0.3 |
| BD46 | EIDD-1931 | Remdesivir | 0.125 | 1 | 2.4 | 0.3 | -1.4 | 0.3 | 3.3 | 0.3 | 1.8 | 0.9 |
| BD46 | EIDD-1931 | Remdesivir | 0.125 | 4 | 53.2 | 3.4 | 53.8 | 3.4 | 54.0 | 3.4 | 53.2 | 3.4 |
| BD46 | EIDD-1931 | Remdesivir | 0.5 | 0.25 | 7.7 | 0.6 | 6.1 | 0.6 | 7.7 | 0.6 | 7.5 | 0.7 |
| BD46 | EIDD-1931 | Remdesivir | 0.5 | 1 | 11.8 | 3.9 | 10.1 | 3.9 | 12.0 | 3.9 | 12.0 | 3.2 |
| BD46 | EIDD-1931 | Remdesivir | 0.5 | 4 | 13.9 | 0.5 | 13.1 | 0.5 | 15.0 | 0.5 | 13.9 | 0.5 |
| AQ28 | EIDD-1931 | Remdesivir | 0.03125 | 0.25 | -1.4 | 0.8 | 0.3 | 0.8 | -1.2 | 0.8 | 1.6 | 0.0 |
| AQ28 | EIDD-1931 | Remdesivir | 0.03125 | 1 | -1.8 | 5.4 | -4.9 | 5.4 | -6.4 | 5.4 | -0.8 | 0.1 |
| AQ28 | EIDD-1931 | Remdesivir | 0.03125 | 4 | 67.2 | 10.1 | 70.1 | 10.1 | 69.7 | 10.1 | 68.5 | 10.0 |
| AQ28 | EIDD-1931 | Remdesivir | 0.125 | 0.25 | -1.9 | 3.3 | -2.7 | 3.3 | -1.8 | 3.3 | 0.6 | 0.3 |
| AQ28 | EIDD-1931 | Remdesivir | 0.125 | 1 | 2.7 | 5.8 | -2.9 | 5.8 | -1.9 | 5.8 | 0.9 | 2.6 |
| AQ28 | EIDD-1931 | Remdesivir | 0.125 | 4 | 83.7 | 7.7 | 83.0 | 7.7 | 83.9 | 7.7 | 82.1 | 6.2 |
| AQ28 | EIDD-1931 | Remdesivir | 0.5 | 0.25 | -29.4 | 5.6 | -28.2 | 5.6 | -29.4 | 5.6 | -25.9 | 7.2 |
| AQ28 | EIDD-1931 | Remdesivir | 0.5 | 1 | -17.2 | 18.7 | -15.0 | 18.4 | -16.1 | 18.7 | -19.6 | 15.5 |
| AQ28 | EIDD-1931 | Remdesivir | 0.5 | 4 | -29.7 | 5.9 | -29.9 | 5.9 | -31.2 | 5.9 | -26.8 | 5.6 |
| AQ23 | EIDD-1931 | Remdesivir | 0.03125 | 0.25 | 8.0 | 6.2 | -0.7 | 6.2 | 1.1 | 6.2 | 3.1 | 2.8 |
| AQ23 | EIDD-1931 | Remdesivir | 0.03125 | 1 | 9.1 | 5.9 | -13.6 | 5.9 | 7.7 | 5.9 | 9.2 | 5.8 |
| AQ23 | EIDD-1931 | Remdesivir | 0.03125 | 4 | -20.5 | 1.8 | -19.4 | 1.8 | -20.4 | 1.8 | -15.6 | 1.5 |
| AQ23 | EIDD-1931 | Remdesivir | 0.125 | 0.25 | 17.2 | 16.6 | 5.6 | 16.6 | 12.2 | 16.6 | 11.9 | 9.9 |
| AQ23 | EIDD-1931 | Remdesivir | 0.125 | 1 | 16.9 | 11.5 | -19.4 | 11.5 | 15.9 | 11.5 | 19.0 | 11.6 |
| AQ23 | EIDD-1931 | Remdesivir | 0.125 | 4 | -7.7 | 4.6 | -6.3 | 4.4 | -7.2 | 4.6 | -9.8 | 3.1 |
| AQ23 | EIDD-1931 | Remdesivir | 0.5 | 0.25 | 6.6 | 1.3 | 2.7 | 1.3 | 5.2 | 1.3 | 5.2 | 1.5 |
| AQ23 | EIDD-1931 | Remdesivir | 0.5 | 1 | 7.3 | 3.3 | -2.7 | 3.3 | 7.0 | 3.3 | 7.8 | 2.8 |
| AQ23 | EIDD-1931 | Remdesivir | 0.5 | 4 | -6.1 | 1.6 | -3.8 | 1.6 | -4.8 | 1.6 | -8.7 | 2.1 |
| Wuhan | Favipiravir | Nirmatrelvir | 0.5 | 0.125 | 2.3 | 0.9 | 3.1 | 1.2 | 2.8 | 0.9 | 2.6 | 1.1 |
| Wuhan | Favipiravir | Nirmatrelvir | 0.5 | 0.5 | 63.9 | 23.3 | 65.2 | 23.3 | 65.9 | 23.3 | 51.8 | 13.3 |
| Wuhan | Favipiravir | Nirmatrelvir | 0.5 | 2 | -25.2 | 9.7 | -25.7 | 9.8 | -25.6 | 9.7 | -21.4 | 9.2 |
| Wuhan | Favipiravir | Nirmatrelvir | 4 | 0.125 | 5.0 | 3.5 | 3.9 | 3.2 | 5.1 | 3.0 | 4.4 | 3.5 |
| Wuhan | Favipiravir | Nirmatrelvir | 4 | 0.5 | 60.9 | 19.2 | 59.6 | 19.0 | 61.6 | 19.8 | 49.2 | 12.0 |
| Wuhan | Favipiravir | Nirmatrelvir | 4 | 2 | -36.1 | 7.3 | -36.3 | 7.2 | -36.2 | 7.2 | -36.7 | 5.3 |
| Wuhan | Favipiravir | Nirmatrelvir | 32 | 0.125 | 2.8 | 4.5 | 2.2 | 4.8 | 3.3 | 4.5 | 3.0 | 4.2 |
| Wuhan | Favipiravir | Nirmatrelvir | 32 | 0.5 | 16.7 | 9.1 | 22.3 | 8.9 | 26.0 | 7.2 | 15.4 | 7.8 |
| Wuhan | Favipiravir | Nirmatrelvir | 32 | 2 | -66.7 | 13.4 | -69.0 | 13.4 | -69.0 | 13.3 | -64.4 | 12.1 |
| BD46 | Favipiravir | EIDD-1931 | 2 | 0.03125 | 1.7 | 0.6 | -0.3 | 0.6 | 0.3 | 0.6 | 1.0 | 0.3 |
| BD46 | Favipiravir | EIDD-1931 | 2 | 0.125 | 0.0 | 0.8 | -1.5 | 0.8 | -0.5 | 0.8 | 0.5 | 0.6 |
| BD46 | Favipiravir | EIDD-1931 | 2 | 0.5 | -8.3 | 5.7 | -7.7 | 5.7 | -8.3 | 5.7 | -4.0 | 2.8 |
| BD46 | Favipiravir | EIDD-1931 | 8 | 0.03125 | 6.2 | 1.3 | 2.6 | 1.3 | 4.1 | 1.3 | 2.2 | 0.6 |
| BD46 | Favipiravir | EIDD-1931 | 8 | 0.125 | 10.1 | 2.5 | 2.8 | 2.5 | 8.1 | 2.5 | 9.1 | 2.5 |
| BD46 | Favipiravir | EIDD-1931 | 8 | 0.5 | 33.3 | 1.9 | 32.7 | 1.9 | 32.9 | 1.9 | 26.3 | 1.4 |
| BD46 | Favipiravir | EIDD-1931 | 32 | 0.03125 | 2.0 | 0.9 | -0.4 | 0.9 | 0.7 | 0.9 | 1.1 | 0.7 |
| BD46 | Favipiravir | EIDD-1931 | 32 | 0.125 | 6.6 | 2.3 | 6.1 | 2.3 | 8.0 | 2.3 | 6.6 | 2.3 |
| BD46 | Favipiravir | EIDD-1931 | 32 | 0.5 | 5.0 | 2.3 | 5.8 | 2.3 | 6.0 | 2.3 | 11.7 | 1.8 |
| AQ28 | Favipiravir | EIDD-1931 | 2 | 0.03125 | 2.6 | 3.1 | 2.7 | 3.1 | 2.1 | 3.1 | 2.6 | 1.7 |
| AQ28 | Favipiravir | EIDD-1931 | 2 | 0.125 | 12.8 | 7.8 | 10.5 | 7.8 | 12.4 | 7.8 | 9.3 | 6.4 |
| AQ28 | Favipiravir | EIDD-1931 | 2 | 0.5 | -14.8 | 1.9 | -13.5 | 1.9 | -14.7 | 1.9 | -11.3 | 2.5 |
| AQ28 | Favipiravir | EIDD-1931 | 8 | 0.03125 | 6.7 | 2.7 | 6.1 | 2.7 | 5.5 | 2.7 | 3.3 | 1.5 |
| AQ28 | Favipiravir | EIDD-1931 | 8 | 0.125 | 11.6 | 4.5 | 8.5 | 4.5 | 10.4 | 4.5 | 8.1 | 5.9 |
| AQ28 | Favipiravir | EIDD-1931 | 8 | 0.5 | 0.5 | 15.0 | 1.4 | 14.4 | 0.8 | 15.0 | -3.7 | 11.8 |
| AQ28 | Favipiravir | EIDD-1931 | 32 | 0.03125 | -1.5 | 2.5 | -11.2 | 2.5 | 0.9 | 2.5 | 0.0 | 2.1 |
| AQ28 | Favipiravir | EIDD-1931 | 32 | 0.125 | 15.9 | 17.9 | -13.2 | 17.9 | 16.0 | 17.9 | 14.8 | 17.7 |
| AQ28 | Favipiravir | EIDD-1931 | 32 | 0.5 | -26.0 | 13.3 | -27.1 | 13.3 | -28.4 | 13.3 | -21.4 | 13.3 |
| AQ23 | Favipiravir | EIDD-1931 | 2 | 0.03125 | 2.3 | 4.1 | -5.9 | 4.1 | -3.4 | 4.1 | -0.2 | 0.9 |
| AQ23 | Favipiravir | EIDD-1931 | 2 | 0.125 | -7.5 | 25.1 | -28.9 | 25.1 | -10.4 | 25.1 | -1.3 | 12.5 |
| AQ23 | Favipiravir | EIDD-1931 | 2 | 0.5 | 19.0 | 9.2 | 18.2 | 9.2 | 18.2 | 9.2 | 17.5 | 7.8 |
| AQ23 | Favipiravir | EIDD-1931 | 8 | 0.03125 | 5.7 | 6.7 | -23.2 | 6.7 | 0.5 | 6.7 | 3.6 | 4.3 |
| AQ23 | Favipiravir | EIDD-1931 | 8 | 0.125 | 8.3 | 27.6 | -7.0 | 27.6 | 12.3 | 27.6 | 12.3 | 21.7 |
| AQ23 | Favipiravir | EIDD-1931 | 8 | 0.5 | 31.2 | 3.8 | 32.3 | 3.8 | 32.3 | 3.8 | 26.0 | 2.8 |
| AQ23 | Favipiravir | EIDD-1931 | 32 | 0.03125 | 19.3 | 3.3 | 16.3 | 3.3 | 16.5 | 3.3 | 14.2 | 5.1 |
| AQ23 | Favipiravir | EIDD-1931 | 32 | 0.125 | 3.1 | 17.4 | 12.1 | 12.4 | 18.5 | 17.4 | 4.1 | 17.3 |
| AQ23 | Favipiravir | EIDD-1931 | 32 | 0.5 | 9.6 | 1.6 | 19.3 | 1.6 | 19.3 | 1.6 | 10.9 | 2.2 |
| Wuhan | Nirmatrelvir | EIDD-1931 | 0.125 | 0.125 | -0.9 | 4.7 | -34.5 | 10.0 | -2.4 | 4.2 | -0.8 | 2.7 |
| Wuhan | Nirmatrelvir | Remdesivir | 0.125 | 0.5 | 4.8 | 1.6 | 3.4 | 1.6 | 4.1 | 1.6 | 2.6 | 0.9 |
| Wuhan | Nirmatrelvir | EIDD-1931 | 0.125 | 0.5 | -32.1 | 23.3 | -28.5 | 24.1 | -31.9 | 23.3 | -25.1 | 17.7 |
| Wuhan | Nirmatrelvir | EIDD-1931 | 0.125 | 2 | -5.2 | 8.4 | -9.3 | 8.3 | -5.1 | 8.5 | -6.9 | 6.3 |
| Wuhan | Nirmatrelvir | Remdesivir | 0.125 | 4 | 25.4 | 6.9 | 9.8 | 8.0 | 25.7 | 6.9 | 25.2 | 6.9 |
| Wuhan | Nirmatrelvir | Remdesivir | 0.125 | 32 | 24.9 | 16.7 | 24.3 | 17.0 | 24.9 | 16.7 | 20.8 | 13.3 |
| Wuhan | Nirmatrelvir | EIDD-1931 | 0.5 | 0.125 | -10.6 | 4.4 | -102.9 | 2.3 | -4.7 | 3.5 | -6.5 | 2.1 |
| Wuhan | Nirmatrelvir | Remdesivir | 0.5 | 0.5 | -3.2 | 4.3 | -10.6 | 5.4 | -3.9 | 4.3 | -1.1 | 2.3 |
| Wuhan | Nirmatrelvir | EIDD-1931 | 0.5 | 0.5 | -42.9 | 16.4 | -41.0 | 16.3 | -44.1 | 16.3 | -33.6 | 11.6 |
| Wuhan | Nirmatrelvir | EIDD-1931 | 0.5 | 2 | 5.1 | 11.6 | -2.1 | 6.7 | 4.5 | 11.9 | -1.2 | 6.7 |
| Wuhan | Nirmatrelvir | Remdesivir | 0.5 | 4 | 32.6 | 8.1 | 0.3 | 9.9 | 41.9 | 7.8 | 32.2 | 6.7 |
| Wuhan | Nirmatrelvir | Remdesivir | 0.5 | 32 | 23.0 | 14.5 | 24.1 | 16.9 | 24.7 | 16.8 | 18.9 | 11.2 |
| Wuhan | Nirmatrelvir | EIDD-1931 | 2 | 0.125 | -13.5 | 5.2 | -13.7 | 5.2 | -14.6 | 5.2 | -14.1 | 5.2 |
| Wuhan | Nirmatrelvir | EIDD-1931 | 2 | 0.5 | -2.7 | 4.3 | -21.1 | 5.4 | -22.4 | 5.2 | -6.8 | 6.5 |
| Wuhan | Nirmatrelvir | Remdesivir | 2 | 0.5 | -27.8 | 8.2 | -28.2 | 8.2 | -27.7 | 8.2 | -27.1 | 6.8 |
| Wuhan | Nirmatrelvir | EIDD-1931 | 2 | 2 | 0.0 | 6.4 | -17.3 | 6.3 | -18.2 | 6.4 | 2.1 | 2.3 |
| Wuhan | Nirmatrelvir | Remdesivir | 2 | 4 | -25.8 | 3.1 | -33.0 | 3.6 | -32.5 | 3.8 | -24.0 | 2.4 |
| Wuhan | Nirmatrelvir | Remdesivir | 2 | 32 | -14.6 | 4.4 | -30.2 | 3.1 | -29.8 | 3.3 | -13.8 | 4.8 |
| BD46 | Nirmatrelvir | EIDD-1931 | 0.0625 | 0.03125 | 2.3 | 1.2 | 0.2 | 1.2 | 0.3 | 1.2 | 0.8 | 1.0 |
| BD46 | Nirmatrelvir | EIDD-1931 | 0.0625 | 0.125 | 10.1 | 2.1 | 10.8 | 2.1 | 10.8 | 2.1 | 7.9 | 1.7 |
| BD46 | Nirmatrelvir | Remdesivir | 0.0625 | 0.25 | -0.8 | 0.9 | 1.1 | 0.9 | -0.7 | 0.9 | 0.0 | 0.2 |
| BD46 | Nirmatrelvir | EIDD-1931 | 0.0625 | 0.5 | 12.1 | 3.0 | 11.4 | 3.0 | 12.2 | 3.0 | 11.0 | 2.6 |
| BD46 | Nirmatrelvir | Remdesivir | 0.0625 | 1 | -2.9 | 0.6 | -1.0 | 0.6 | -1.3 | 0.6 | -0.9 | 0.0 |
| BD46 | Nirmatrelvir | Remdesivir | 0.0625 | 4 | 6.6 | 1.7 | 8.4 | 1.7 | 8.4 | 1.7 | 4.4 | 1.1 |
| BD46 | Nirmatrelvir | EIDD-1931 | 0.25 | 0.03125 | 1.0 | 2.1 | -1.6 | 2.1 | -1.0 | 2.1 | 0.9 | 1.0 |
| BD46 | Nirmatrelvir | EIDD-1931 | 0.25 | 0.125 | 6.8 | 1.6 | 7.1 | 1.6 | 7.1 | 1.6 | 9.2 | 1.3 |
| BD46 | Nirmatrelvir | Remdesivir | 0.25 | 0.25 | 1.6 | 0.3 | 0.0 | 0.3 | 1.0 | 0.3 | -0.2 | 0.2 |
| BD46 | Nirmatrelvir | EIDD-1931 | 0.25 | 0.5 | 8.0 | 1.0 | 6.4 | 1.0 | 8.1 | 1.0 | 9.4 | 1.0 |
| BD46 | Nirmatrelvir | Remdesivir | 0.25 | 1 | -2.4 | 0.1 | -3.0 | 0.1 | -3.0 | 0.1 | -1.2 | 0.0 |
| BD46 | Nirmatrelvir | Remdesivir | 0.25 | 4 | 15.9 | 0.7 | 15.4 | 0.7 | 15.4 | 0.7 | 13.7 | 4.4 |
| BD46 | Nirmatrelvir | EIDD-1931 | 1 | 0.03125 | 4.3 | 1.4 | 1.7 | 1.4 | 2.2 | 1.4 | 3.0 | 1.4 |
| BD46 | Nirmatrelvir | EIDD-1931 | 1 | 0.125 | 24.4 | 0.2 | 24.7 | 0.2 | 24.7 | 0.2 | 23.7 | 0.5 |
| BD46 | Nirmatrelvir | Remdesivir | 1 | 0.25 | -1.5 | 0.5 | -1.9 | 0.5 | -1.4 | 0.5 | 0.1 | 0.0 |
| BD46 | Nirmatrelvir | EIDD-1931 | 1 | 0.5 | 14.3 | 2.7 | 12.4 | 2.7 | 14.3 | 2.7 | 13.2 | 2.6 |
| BD46 | Nirmatrelvir | Remdesivir | 1 | 1 | 5.0 | 1.8 | 2.9 | 1.3 | 5.7 | 1.8 | 5.5 | 2.1 |
| BD46 | Nirmatrelvir | Remdesivir | 1 | 4 | 80.4 | 4.1 | 81.0 | 4.1 | 81.0 | 4.1 | 80.9 | 4.1 |
| AQ28 | Nirmatrelvir | EIDD-1931 | 0.0625 | 0.03125 | -4.2 | 2.6 | -2.8 | 2.6 | -3.9 | 2.6 | 0.3 | 1.5 |
| AQ28 | Nirmatrelvir | EIDD-1931 | 0.0625 | 0.125 | -1.0 | 3.5 | -2.1 | 3.5 | -0.8 | 3.5 | 0.8 | 0.8 |
| AQ28 | Nirmatrelvir | Remdesivir | 0.0625 | 0.25 | -7.1 | 1.4 | -0.6 | 1.4 | -6.9 | 1.4 | 2.6 | 0.7 |
| AQ28 | Nirmatrelvir | EIDD-1931 | 0.0625 | 0.5 | -26.6 | 7.8 | -25.4 | 7.8 | -26.6 | 7.8 | -24.0 | 7.5 |
| AQ28 | Nirmatrelvir | Remdesivir | 0.0625 | 1 | -8.0 | 4.7 | -1.7 | 4.7 | -8.0 | 4.7 | 2.4 | 0.5 |
| AQ28 | Nirmatrelvir | Remdesivir | 0.0625 | 4 | 60.1 | 8.9 | 67.4 | 8.9 | 66.1 | 8.9 | 65.0 | 8.5 |
| AQ28 | Nirmatrelvir | EIDD-1931 | 0.25 | 0.03125 | 2.7 | 3.3 | 0.0 | 3.3 | -1.1 | 3.3 | -1.4 | 2.1 |
| AQ28 | Nirmatrelvir | EIDD-1931 | 0.25 | 0.125 | 8.0 | 9.4 | 2.8 | 9.4 | 4.1 | 9.4 | 5.5 | 7.8 |
| AQ28 | Nirmatrelvir | Remdesivir | 0.25 | 0.25 | 6.7 | 4.5 | 1.6 | 4.5 | 3.0 | 4.5 | -2.6 | 1.4 |
| AQ28 | Nirmatrelvir | EIDD-1931 | 0.25 | 0.5 | -23.1 | 16.2 | -21.2 | 16.0 | -22.3 | 16.2 | -23.5 | 13.2 |
| AQ28 | Nirmatrelvir | Remdesivir | 0.25 | 1 | 1.5 | 3.2 | -3.9 | 3.2 | -2.3 | 3.2 | -2.3 | 1.6 |
| AQ28 | Nirmatrelvir | Remdesivir | 0.25 | 4 | 78.4 | 6.9 | 74.6 | 6.9 | 74.9 | 6.9 | 75.3 | 5.5 |
| AQ28 | Nirmatrelvir | EIDD-1931 | 1 | 0.03125 | -10.8 | 12.4 | -8.9 | 12.4 | -10.0 | 12.4 | -1.4 | 7.3 |
| AQ28 | Nirmatrelvir | EIDD-1931 | 1 | 0.125 | 32.1 | 31.4 | 31.5 | 31.4 | 32.3 | 31.4 | 33.3 | 30.5 |
| AQ28 | Nirmatrelvir | Remdesivir | 1 | 0.25 | -3.1 | 7.9 | -3.2 | 7.8 | -2.9 | 7.9 | 0.4 | 4.3 |
| AQ28 | Nirmatrelvir | EIDD-1931 | 1 | 0.5 | -25.8 | 6.9 | -24.8 | 6.9 | -26.0 | 6.9 | -27.3 | 6.4 |
| AQ28 | Nirmatrelvir | Remdesivir | 1 | 1 | 12.1 | 17.2 | 11.5 | 17.2 | 12.1 | 17.2 | 12.7 | 16.4 |
| AQ28 | Nirmatrelvir | Remdesivir | 1 | 4 | 85.7 | 6.8 | 89.5 | 6.8 | 90.0 | 6.8 | 84.9 | 6.1 |
| AQ23 | Nirmatrelvir | EIDD-1931 | 0.0625 | 0.03125 | -13.7 | 6.7 | -19.4 | 6.7 | -19.1 | 6.7 | -0.6 | 0.0 |
| AQ23 | Nirmatrelvir | EIDD-1931 | 0.0625 | 0.125 | 4.8 | 23.2 | -19.3 | 23.2 | 5.8 | 23.2 | 3.8 | 13.8 |
| AQ23 | Nirmatrelvir | Remdesivir | 0.0625 | 0.25 | 4.9 | 5.1 | 6.0 | 5.1 | -1.0 | 5.1 | 7.5 | 1.5 |
| AQ23 | Nirmatrelvir | EIDD-1931 | 0.0625 | 0.5 | 12.6 | 2.6 | 12.9 | 2.6 | 12.9 | 2.6 | 9.5 | 1.6 |
| AQ23 | Nirmatrelvir | Remdesivir | 0.0625 | 1 | 1.7 | 3.8 | 5.2 | 3.8 | 1.1 | 3.8 | 9.6 | 1.5 |
| AQ23 | Nirmatrelvir | Remdesivir | 0.0625 | 4 | -18.4 | 3.6 | -16.7 | 3.6 | -18.3 | 3.6 | -15.2 | 2.4 |
| AQ23 | Nirmatrelvir | EIDD-1931 | 0.25 | 0.03125 | -10.4 | 14.9 | -28.1 | 14.9 | -15.8 | 14.9 | -0.2 | 1.7 |
| AQ23 | Nirmatrelvir | EIDD-1931 | 0.25 | 0.125 | -7.7 | 17.9 | -54.8 | 17.9 | -6.3 | 17.9 | 0.2 | 12.1 |
| AQ23 | Nirmatrelvir | Remdesivir | 0.25 | 0.25 | 26.2 | 7.9 | 3.2 | 7.9 | 9.6 | 7.9 | 6.5 | 6.5 |
| AQ23 | Nirmatrelvir | EIDD-1931 | 0.25 | 0.5 | 3.3 | 1.0 | 3.6 | 1.0 | 3.6 | 1.0 | 4.7 | 0.8 |
| AQ23 | Nirmatrelvir | Remdesivir | 0.25 | 1 | 33.1 | 6.3 | -8.9 | 6.3 | 17.3 | 6.3 | 23.3 | 4.9 |
| AQ23 | Nirmatrelvir | Remdesivir | 0.25 | 4 | -11.3 | 1.3 | -12.9 | 1.3 | -11.6 | 1.3 | -12.3 | 0.8 |
| AQ23 | Nirmatrelvir | EIDD-1931 | 1 | 0.03125 | -13.0 | 5.7 | -13.9 | 5.7 | -14.1 | 5.7 | -6.5 | 2.8 |
| AQ23 | Nirmatrelvir | EIDD-1931 | 1 | 0.125 | -5.7 | 7.1 | 0.2 | 6.7 | 0.4 | 7.1 | -5.5 | 6.9 |
| AQ23 | Nirmatrelvir | Remdesivir | 1 | 0.25 | -15.3 | 5.4 | -36.4 | 5.4 | -16.6 | 5.4 | -7.9 | 2.7 |
| AQ23 | Nirmatrelvir | EIDD-1931 | 1 | 0.5 | -6.4 | 0.6 | 9.2 | 0.6 | 9.2 | 0.6 | -7.5 | 0.5 |
| AQ23 | Nirmatrelvir | Remdesivir | 1 | 1 | -1.5 | 2.4 | -21.6 | 2.4 | -1.7 | 2.4 | -0.6 | 1.6 |
| AQ23 | Nirmatrelvir | Remdesivir | 1 | 4 | -9.4 | 1.8 | -9.4 | 1.8 | -8.1 | 1.8 | -11.5 | 1.4 |

**Supplemental Table S4**. Cytotoxicity combination scores for different drug combinations against ancestral SARS-CoV-2 strain in Vero E6 cells.

| **Combination** | **conc1** | **conc2** | **Cytotoxicity synergy score** | **synergy score sd** | **response** | **response sd** |
| --- | --- | --- | --- | --- | --- | --- |
| Nirmatrelvir - Remdesivir | 0.125 | 32 | 3.9 | 11.0 | 0.8 | 0.0 |
| Nirmatrelvir - Remdesivir | 0.5 | 32 | <0 | n/a | 29.7 | 0.0 |
| Nirmatrelvir - Remdesivir | 2 | 32 | <0 | n/a | -3.4 | 2.1 |
| Nirmatrelvir - Remdesivir | 0.125 | 4 | <0 | n/a | 23.4 | 0.0 |
| Nirmatrelvir - Remdesivir | 0.5 | 4 | <0 | n/a | 4.4 | 5.4 |
| Nirmatrelvir - Remdesivir | 2 | 4 | <0 | n/a | -30.0 | 35.7 |
| Nirmatrelvir - Remdesivir | 0.125 | 0.5 | <0 | n/a | -11.6 | 13.7 |
| Nirmatrelvir - Remdesivir | 0.5 | 0.5 | <0 | n/a | -18.2 | 17.4 |
| Nirmatrelvir - Remdesivir | 2 | 0.5 | <0 | n/a | 19.2 | 0.0 |
| Nirmatrelvir - EIDD-1931 | 2 | 0.125 | <0 | n/a | 0.8 | 0.0 |
| Nirmatrelvir - EIDD-1931 | 2 | 0.5 | <0 | n/a | -18.9 | 0.0 |
| Nirmatrelvir - EIDD-1931 | 2 | 2 | <0 | n/a | -5.4 | 0.0 |
| Nirmatrelvir - EIDD-1931 | 0.5 | 0.125 | <0 | n/a | 3.9 | 15.0 |
| Nirmatrelvir - EIDD-1931 | 0.5 | 0.5 | <0 | n/a | -14.9 | 0.0 |
| Nirmatrelvir - EIDD-1931 | 0.5 | 2 | <0 | n/a | -10.5 | 22.3 |
| Nirmatrelvir - EIDD-1931 | 0.125 | 0.125 | <0 | n/a | -1.4 | 51.9 |
| Nirmatrelvir - EIDD-1931 | 0.125 | 0.5 | <0 | n/a | 2.2 | 14.0 |
| Nirmatrelvir - EIDD-1931 | 0.125 | 2 | <0 | n/a | -54.7 | 0.0 |
| EIDD-1931 - Remdesivir | 0.125 | 32 | <0 | n/a | 0.8 | 0.0 |
| EIDD-1931 - Remdesivir | 0.5 | 32 | <0 | n/a | 29.7 | 0.0 |
| EIDD-1931 - Remdesivir | 2 | 32 | 2.6 | 5.7 | -3.4 | 2.1 |
| EIDD-1931 - Remdesivir | 0.125 | 4 | <0 | n/a | 23.4 | 0.0 |
| EIDD-1931 - Remdesivir | 0.5 | 4 | <0 | n/a | -18.9 | 0.0 |
| EIDD-1931 - Remdesivir | 2 | 4 | 15.3 | 8.3 | -30.9 | 24.5 |
| EIDD-1931 - Remdesivir | 0.125 | 0.5 | <0 | n/a | -19.3 | 10.2 |
| EIDD-1931 - Remdesivir | 0.5 | 0.5 | <0 | n/a | -20.4 | 26.2 |
| EIDD-1931 - Remdesivir | 2 | 0.5 | <0 | n/a | -5.4 | 0.0 |

**Supplementary Figures S1-S5**

**
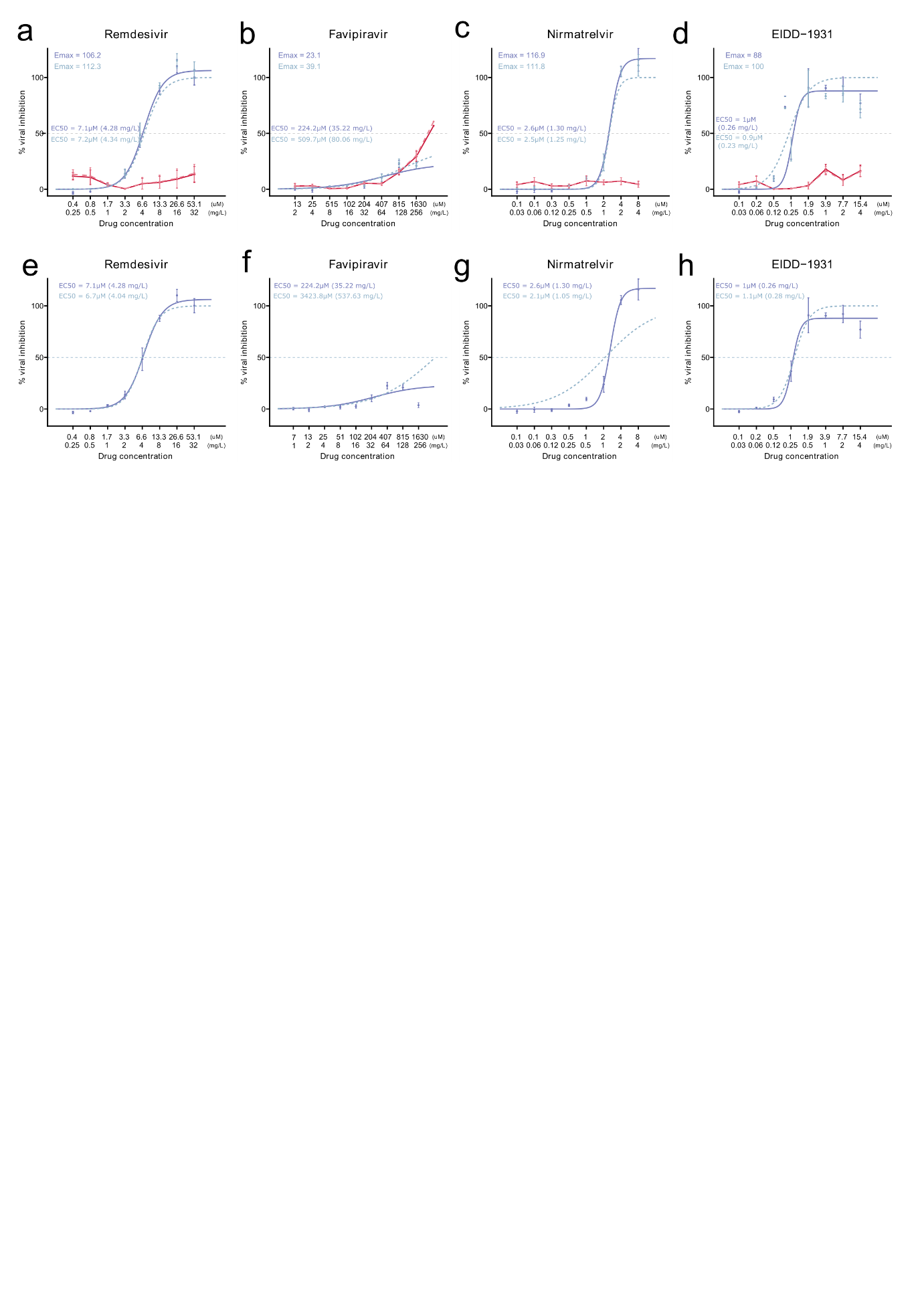
**

**Supplementary Fig. S1.** (a-d) The dose-response curves for Remdesivir (a), Favipiravir (b), Nirmatrelvir (c), and EIDD-1931 (d) showing % viral inhibition (blue) and cytotoxicity (red) before (dashed lines) and after (solid lines) accounting for the 96 well plate edge effect (e-h) The dose-response curves with Emax estimated (solid lines) or fixed (dashed lines) at various drug concentrations for Remdesivir (e), Favipiravir (f), Nirmatrelvir (g), and EIDD-1931 (h) EC50 is given, and error bars represent the standard deviation (n=3-6).

**
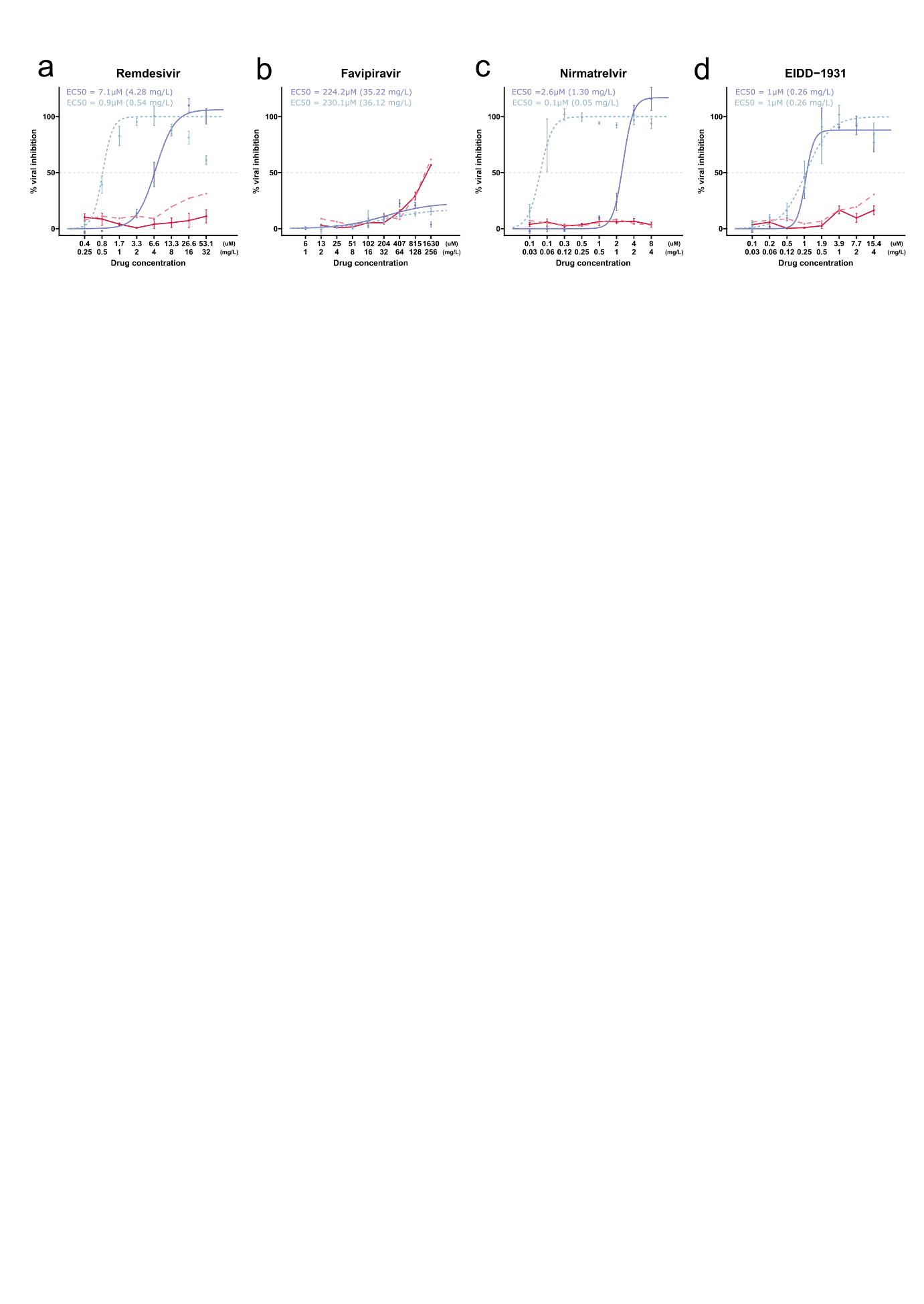
**

**Supplementary Fig. S2.** The dose-response curves for Remdesivir (a), Favipiravir (b), Nirmatrelvir (c), and EIDD-1931 (d) showing % viral inhibition (blue) and cytotoxicity (red) in the absence (solid lines) or presence of (dashed lines) of 0.25 mg/L (0.5 μM) Pgp-inhibitor CP-100356 at various drug concentrations. EC50 is given, and error bars represent the standard deviation (n=3-6).


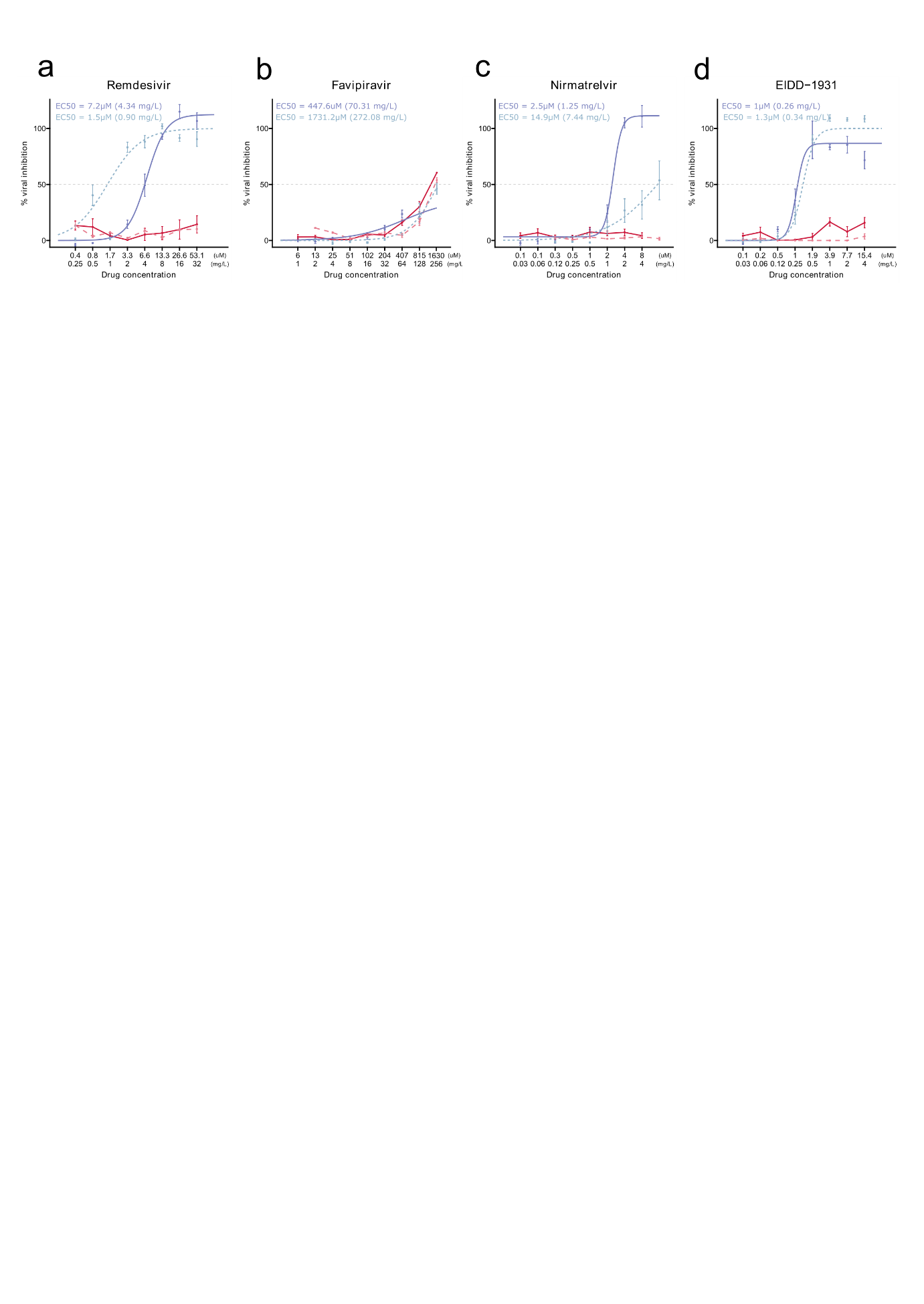


**Supplementary Fig. S3.** (a) The drug-response curves at 72hrs for Remdesivir (a), Favipiravir (b), Nirmatrelvir (c), and EIDD-1931 (d) showing % cytotoxicity (red lines) and Viral toxicity (blue lines) at various drug concentrations for Calu-3 cells (dashed lines) and Vero (solid lines).


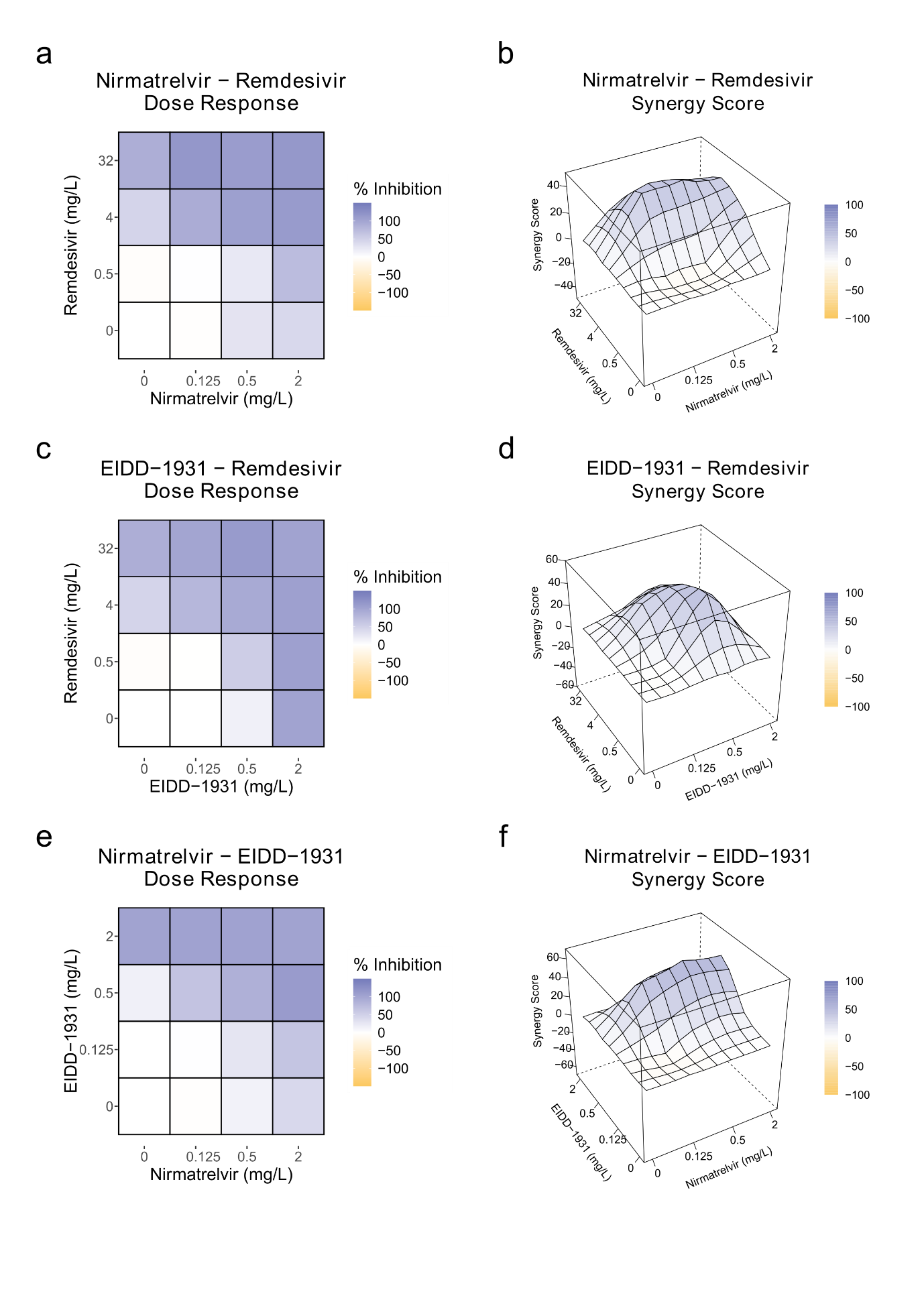


**Supplementary Fig. S4. Antiviral drug combination efficacy in Calu-3 cells.** Heat maps represent viral inhibition and 3D matrices represent Bliss synergy scores for the same respective drug combinations for Nirmatrelvir + Remdesivir (a,b), Nirmatrelvir + EIDD-1931 (c,d), and EIDD-1931+ Remdesivir (e,f) combinations. Inhibition colour scales represented as blue for high and white for low inhibition. Potential synergistic effects (0-100) are depicted in blue, no effect (=0) in white, and possible antagonistic effects (-100-0) in yellow.  The colour scales are given by the distance from 0. Showing mean scores (n=6).


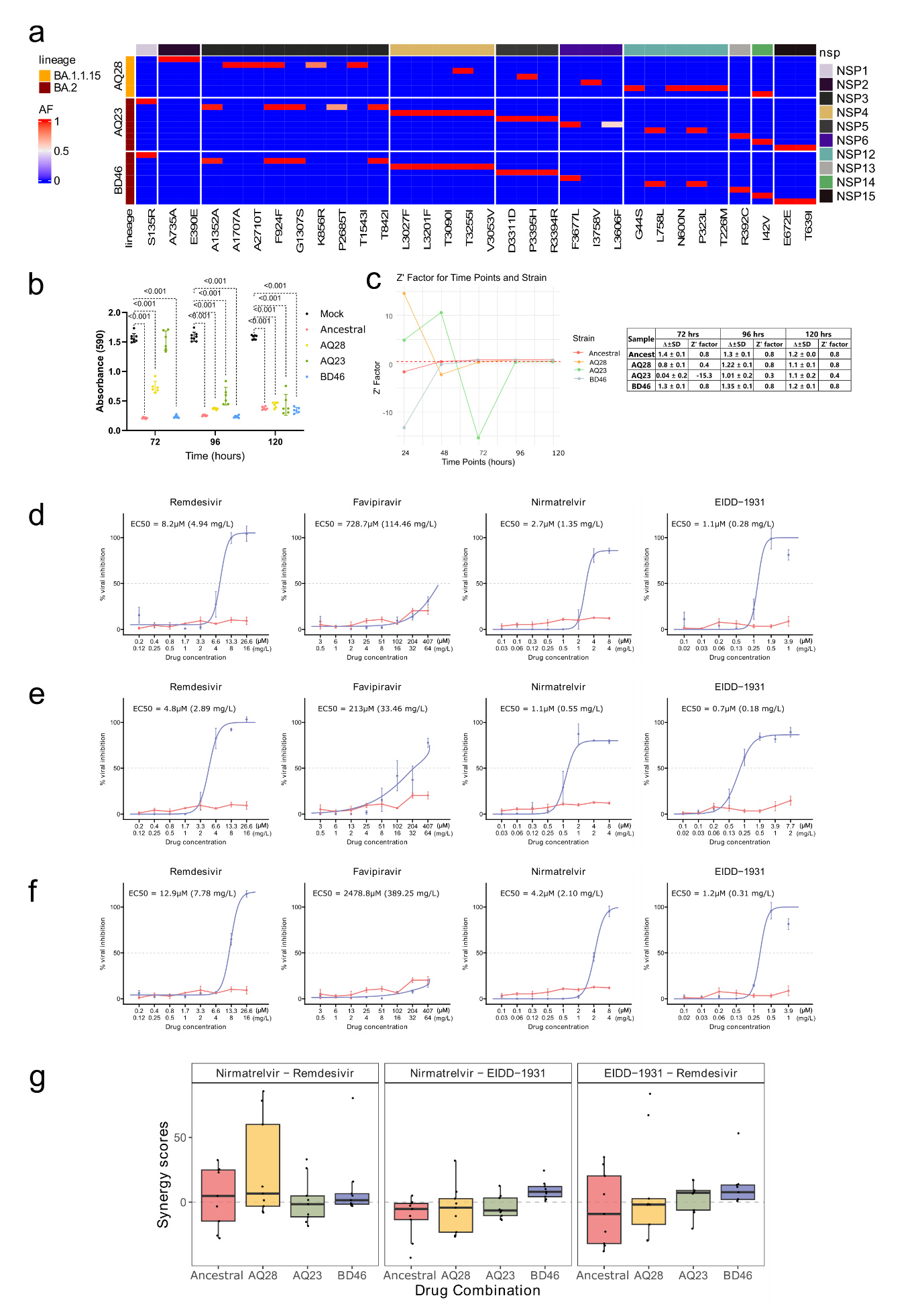


**Supplementary Fig. S5.** (a) Heat map showing the non-structural protein landscape of consensus mutations within different clinical isolates (AQ28, AQ23, BD46) compared to the ancestral strain, allele frequency is represented by blue-red colour scale. (b) the difference in absorbance at 595nm for mock-infected vs infected wells, at different timepoints for all viruses (c) Z-Factor for the assay at different timepoints for each strain, with the numerical coefficients presented on the right. The drug-response curves at 96 hrs for AQ28 (d), AQ23 (e) and BD46 (f) for Remdesivir, Favipiravir, Nirmatrelvir and EIDD-1931, showing % cytotoxicity (red lines) and Viral toxicity (blue lines) at various drug concentrations. Error bars show SE (n=3). (g) Box plot of Bliss synergy scores for nirmatrelvir-remdesivir, nirmatrelvir-EIDD-1931, and EIDD-1931-remdesivir combinations for the different strains. The line in the box shows the median and edges of the box are the first and third quantiles. The whiskers end at the largest/smallest value within the 1.5 interquartile range (IQR = Q3- Q1). Raw data are shown as points.
